# Exploration of biodistribution of mRNA-delivering lipid nanoparticles in the neonatal mouse inner ear

**DOI:** 10.1016/j.ijpx.2026.100675

**Published:** 2026-09-25

**Authors:** Dongsik Park, Byeonghyeon Lee, Ji-Hyun Ma, Yeeun Kim, Jong Su Hwang, Hae Woo Lee, Myung Hoon Yoo, Un-Kyung Kim, Tae-Jun Kwon

**Affiliations:** a Drug Manufacturing Center, Daegu-Gyeongbuk Medical Innovation Foundation (K-MEDI hub), Daegu, Republic of Korea; b New Drug Development Center, Daegu-Gyeongbuk Medical Innovation Foundation (K-MEDI hub), Daegu, Republic of Korea; c Advanced Bio-Resource Research Center, Kyungpook National University, Daegu, Republic of Korea; d Department of Biology, College of Natural Sciences, Kyungpook National University, Daegu, Republic of Korea; e School of Life Sciences, BK21 plus KNU Creative BioResearch Group, Kyungpook National University, Daegu, Republic of Korea; f Department of Otorhinolaryngology-Head and Neck Surgery, Kyungpook National University Hospital, School of Medicine, Kyungpook National University, Daegu, Republic of Korea; g Preclinical Research Center, Daegu-Gyeongbuk Medical Innovation Foundation (K-MEDI hub), Daegu, Republic of Korea

**Keywords:** Lipid nanoparticle, Hearing loss, Gene therapy, Gene transduction, mRNA delivery

## Abstract

Hearing loss affects millions worldwide, yet hearing aids and cochlear implants have major limitations. mRNA-based gene therapy could address the underlying molecular causes, but lipid nanoparticle (LNP) delivery in the inner ear remains underexplored and its biodistribution has not been systematically characterized. Here we establish and scale up an mRNA-encapsulated LNP formulation using a design-of-experiments (DoE) approach built on predefined critical quality attributes, and map the route-dependent distribution of reporter mRNA expression in the neonatal mouse cochlea. In HEI-OC1 auditory cells, mRNA-loaded LNPs produced a higher proportion of reporter-expressing cells and a more uniform distribution of expression than the cationic reagent Lipofectamine at a matched mRNA dose, while total reporter protein was comparable and metabolic viability was not reduced. We then compared cochlear distribution following round window membrane (RWM) delivery *versus* scala media administration. RWM delivery produced widespread reporter expression in perilymph-exposed compartments—the spiral ligament, the scala vestibuli and scala tympani regions, the stria vascularis and the spiral ganglion region—with no detectable expression in hair cells. Scala media injection produced a more restricted pattern, largely confined to the cuticular plate of inner and outer hair cells and part of the outer sulcus, with no expression in the spiral ganglion region or auditory nerve, indicating that this route does not distribute beyond the endolymphatic compartment. These results define the cochlear compartments accessible to LNP-mediated mRNA delivery and the dependence of that access on administration route, and provide the delivery map required for the design of LNP-based mRNA strategies in the inner ear.

## Highlights

• Optimized LNP formulation enables efficient mRNA delivery to inner ear tissues.
• Compared round window and scala media injection for cochlear gene expression.
• Scala media injection achieved localized expression near sensory epithelium.
• LNP-mRNA platform offers promising strategy for inner ear gene therapy.

## Graphical abstract

**Figure:**
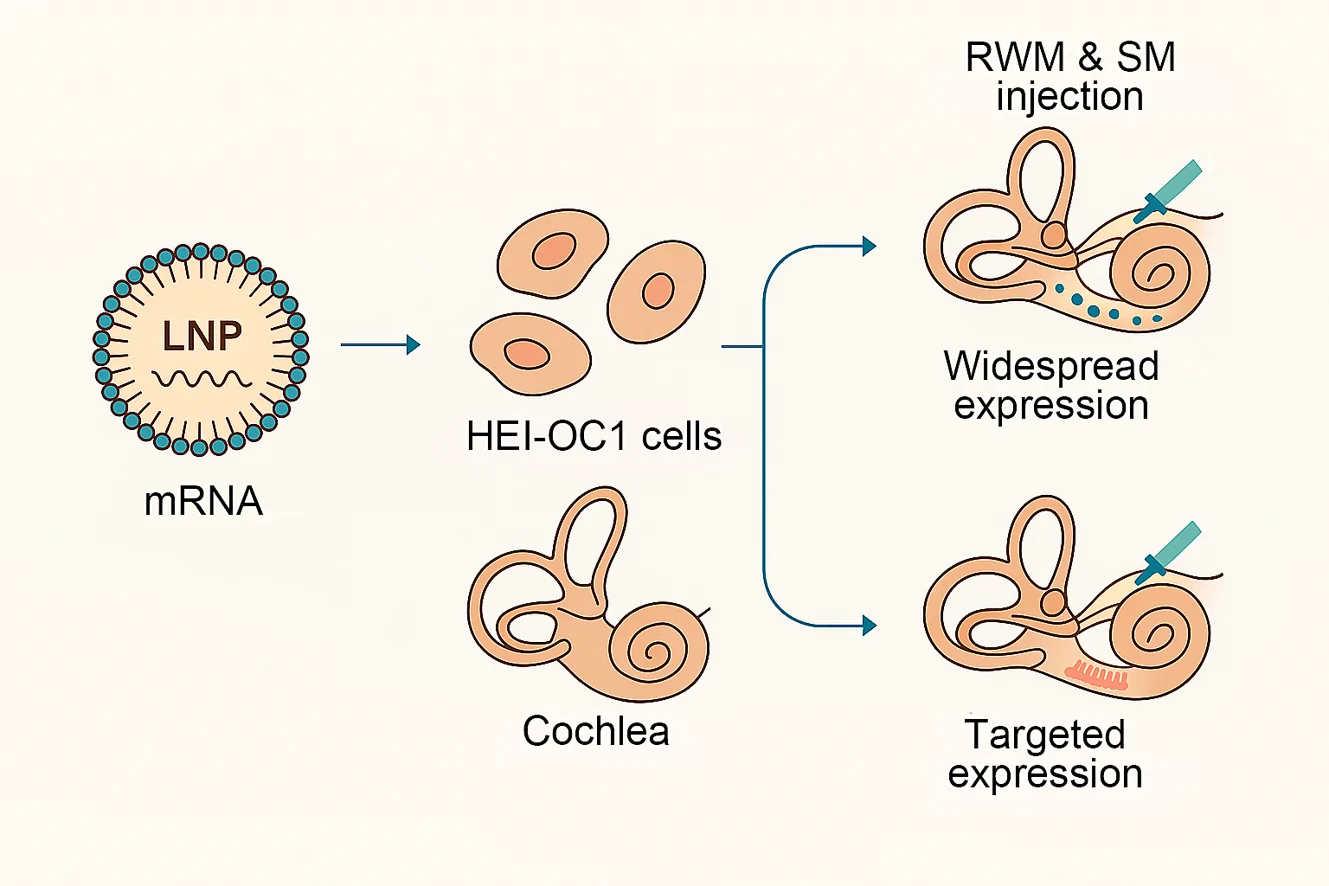

## 1 Introduction

Hearing loss represents a significant disability affecting millions globally, with serious repercussions for communication, quality of life, and overall well-being (Amariutei et al., 2023). However, despite advancements in medical devices such as hearing aids and cochlear implants, these interventions fail to address the underlying causes of auditory impairment, reducing therapeutic efficacy as cochlear degeneration progresses (Gadenstaetter et al., 2025). Furthermore, these devices do not replicate the auditory experience of a normal ear, particularly in terms of speech clarity in noisy environments (Lee and Park, 2018). In this context, gene therapy, which involves introducing deficient genes, replacing mutated genes, or knocking out mutated genes, has emerged as a promising strategy for restoring hearing by directly targeting the molecular defects associated with auditory dysfunction (Ryu et al., 2018). Hence, gene therapy can potentially transform the treatment landscape for hearing loss, offering innovative avenues for restoring or preserving auditory capabilities.

Various genetic materials, including mRNA, oligonucleotides, and plasmids, have been investigated for gene therapy. Among these, mRNA-based therapeutic approaches have gained considerable attention. Unlike DNA-based therapies, mRNA does not necessitate nuclear translocation for functional expression and thus exhibits higher transfection efficiency (Wang et al., 2023). Once in the cytoplasm, mRNA can be translated into sequence-specific therapeutic proteins (Wang et al., 2023; Wang et al., 2021). Additionally, since mRNA does not require genomic integration, the risk of genetic mutagenesis is mitigated (Wang et al., 2021). Therefore, coupled with its rapid design and cell-free production capabilities, mRNA has emerged as a promising therapeutic agent for various diseases (Wang et al., 2021).

Developing efficient delivery systems is crucial to implement mRNA-based therapeutic strategies. Although adeno-associated virus (AAV)-based gene delivery systems have garnered interest, these systems are associated with potential risks, including insertional mutagenesis and immune responses that may result in adverse side effects (Nault et al., 2015). Consequently, non-viral vector-based gene delivery systems are increasingly favored due to their enhanced safety profiles and reduced risk of genomic integration. Among various non-viral gene delivery nanoparticles, lipid nanoparticles (LNPs) have emerged as a leading gene delivery platform in clinical trials, owing to their capacity to protect mRNA from enzymatic degradation, facilitate cellular uptake, and promote endosomal escape (Tenchov et al., 2021; Pozzi and Caracciolo, 2023). The recent success of LNP-encapsulated mRNA vaccines for COVID-19 has further underscored the scalability, safety, and efficacy of this technology (Pozzi and Caracciolo, 2023; Zhang et al., 2023). Building on these advancements, LNPs represent a promising modality for delivering therapeutic mRNA to target tissues, including the intricate structures of the inner ear. Despite this potential, the biomedical application of LNP-based mRNA delivery to the inner ear remains exceedingly rare. To the best of our knowledge, no studies have previously explored the biodistribution of LNPs in the inner ear.

While AAV-mediated inner-ear gene therapy has recently reached clinical reality—demonstrated by the regulatory approval of a dual-vector AAV system for OTOF-related deafness (DFNB9) that established proof of principle for human cochlear gene transfer (Qi et al., 2025)—non-viral LNP platforms offer complementary properties that merit evaluation for specific therapeutic applications: First, LNP-mediated mRNA expression is transient, which aligns well with therapeutic strategies or gene-editing tools where temporary expression minimizes potential off-target effects (Zak and Zangi, 2025). Second, LNP-based delivery is not constrained by structural packaging limits (Zhang et al., 2023; Levine et al., 2026), whereas delivering large genes such as OTOF *via* AAV is capacity-limited and requires architectural modifications including split-and-recombine dual-AAV systems (Grieger and Samulski, 2005; McClements and MacLaren, 2017). Third, the absence of viral capsids reduces the risk of robust neutralizing antibody responses, thereby supporting the potential feasibility of repeated administration (Kenjo et al., 2021). Finally, LNP manufacturing is cell-free and largely sequence-agnostic, offering operational advantages in scalability and standardization (Wang et al., 2021; Kowalski et al., 2019). From these potential features of LNPs, this study evaluates their capacity for mRNA delivery within the inner ear, exploring their utility as a non-viral delivery system.

Thus, this study set out to establish an mRNA-LNP formulation suitable for inner ear administration and to determine where that formulation distributes within the cochlea. We did not set out to demonstrate therapeutic correction of hearing loss, and the experiments reported here do not test it. To achieve our aim, we established an appropriate LNP formulation by utilizing the design of experiments (DoE) approach and leveraging critical quality attributes (CQAs) and critical formulation/process parameters (CPPs). Subsequently, we assessed the distribution of LNP-mediated mRNA transfection in an auditory cell line and in cochlear explants. Finally, we mapped the biodistribution of LNPs in the inner ear *in vivo* following administration by two distinct routes. These findings provide a spatial map of LNP biodistribution in the inner ear and a foundation for future investigation of payload-specific therapeutic applications.

## 2 Materials and methods

### 2.1 Materials

The materials used comprised: 9-heptadecanyl 8-[(2-hydroxyethyl)[6-oxo-6-(undecyloxy)hexyl]amino]octanoate (BroadPharm, SM-102, USA) and [(4-hydroxybutyl)azanediyl]di(hexane-6,1-diyl) bis(2-hexyldecanoate) (BroadPharm, ALC-0315, USA); cholesterol (≥99%) and sucrose (BioXtra, Sigma-Aldrich, S7903, Germany); DSPC (1,2-distearoyl-sn-glycero-3-phosphocholine, ≥99%) (Avanti Research, A80365, USA) and DMG-PEG 2000 (1,2-dimyristoyl-rac-glycero-3-methoxypolyethylene glycol-2000) (Avanti Research, A80151, USA); enhanced green fluorescence protein (EGFP)-encoding mRNA (Trilink, L-7601, USA) and mCherry-encoding mRNA (Trilink, L-7203, USA); 10× phosphate-buffered saline (PBS) buffer, 20× TE buffer, Quant-iT Ribogreen RNA reagent, and Dialysis Cassettes (Thermo Fisher Scientific, R11490, USA); ethanol (Korean Pharmacopoeia (KP) grade, Samchun, 000E1095, Republic of Korea); DEPC-treated water (Bioneer, C-9030, Republic of Korea).

### 2.2 Manufacturing LNP

LNPs were produced using the solvent-injection method followed by dialysis (Evers et al., 2018). Lipids were dissolved in ethanol at molar ratios of 50:10:38.5:1.5 (ionizable lipid/DSPC/cholesterol/PEG2000-DMG), as utilized in clinical formulations (Hald Albertsen et al., 2022). This composition was fixed deliberately rather than optimized, so that the effect of the manufacturing process parameters could be evaluated independently and the formulation retained within a lipid composition for which human safety data already exist. The stock mRNA (1 mg/mL) was diluted in sodium acetate buffer (pH 4.0) with DEPC-treated water to achieve the desired concentrations. These solutions were mixed at high speed (up to 3000 rpm) in small volumes (200 μL) or using a microfluidic device (enParticle, Republic of Korea). Following mixing, formulations were dialyzed against PBS (pH 7.4) in a dialysis cassette for 24 h. Post-dialysis, LNPs were stored at 4 °C and utilized within two weeks. Particle size and polydispersity index (PDI) were determined *via* dynamic light scattering (Zetasizer Nano ZS90, Malvern Panalytical, UK).

### 2.3 Design and execution of 3^2^-full factorial experimental runs for LNP preparation

LNPs were prepared *via* the solvent injection method, employing a 3^2^-full factorial design for establishing a suitable formulation. The aqueous-to-ethanol solution ratio (*v*/v) (X_1_) and the N/P ratio between the ionizable lipid in LNP and mRNA (X_2_) were selected as independent manufacturing factors and investigated at low, medium, and high levels. The response variables included particle size (Y_1_, nm), PDI of LNP (Y_2_), and encapsulation efficiency (EE) (Y_3_, %). The response values obtained from all nine factorial runs were analyzed using Design Expert software (Design Expert 12, Stat-Ease, USA). Three-dimensional surface, contour, overlay plots were generated and analyzed using the same software.

The EE (%) was determined using the Quant-iT RiboGreen RNA reagent, following a previously described method with slight modifications (Akinc et al., 2009; Kontturi et al., 2019). Intact LNP samples were diluted in 1× TE buffer alone to assess external (unencapsulated) RNA, whereas total RNA content was determined following membrane disruption by diluting the formulation 10-fold in 1× TE buffer containing 0.5% (*v*/v) Triton X-100 (Sigma-Aldrich, T8787, USA). After incubation with the RiboGreen dye, fluorescence was detected on a microplate reader (λ_ex_ = 480 nm, λ_em_ = 520 nm). EE was defined as the percentage of encapsulated RNA relative to the total RNA concentration.

To preliminarily screen the transfection efficiencies of the nine LNP formulations, NIH/3T3 cells were utilized. GFP expression in NIH/3T3 cells for each LNP was determined as follows. NIH/3T3 cells were seeded in 96-well plates at a density of 1 × 10^4^ cells per well and incubated for 24 h. Subsequently, the cells were treated with each LNP formulation adjusted to an equivalent amount of RNA and incubated for an additional 24 h. The culture medium was then aspirated and replaced with PBS, and cellular fluorescence was measured using a microplate reader (λ_ex_ = 480 nm, λ_em_ = 520 nm). To quantify LNP-mediated GFP expression, the baseline autofluorescence of untreated control cells was subtracted from the fluorescence intensity of each LNP-treated sample, and the mean fluorescence values were calculated (*n* = 4).

### 2.4 Animals

C57BL/6 male mice were obtained from Hyochang Science (Republic of Korea). Organotypic cochlear explant cultures were prepared from postnatal day 3 (P3) mice, whereas *in vivo* round window membrane (RWM) injections were performed in neonatal mice at postnatal day 0–2 (P0–P2). Animals were maintained under standard laboratory conditions with free access to food and water under a 12 h light/12 h dark cycle. The animal experiments were conducted between May 2022 and May 2024. Sample sizes for each experiment are indicated in the corresponding figure legends. Animals were allocated to experimental groups before the experiments. Pups were monitored daily after surgery for body weight, color, spontaneous movement, feeding and maternal acceptance; predefined humane endpoints were failure of maternal acceptance, absence of a milk spot, or loss or failure to gain body weight, and any animal meeting these criteria was euthanized. The study was designed to evaluate the physicochemical properties, transfection efficiency, and biodistribution of mRNA-loaded lipid nanoparticles using *in vitro*, *ex vivo*, and *in vivo* models.

All animal procedures were approved by the Institutional Animal Care and Use Committee (IACUC) of Kyungpook National University (Approval No. 2022–0147) and were conducted in accordance with the National Institutes of Health Guide for the Care and Use of Laboratory Animals and the ARRIVE 2.0 guidelines.

### 2.5 Cell culture and transfection

HEI-OC1 auditory cells were cultured for 16 h in Dulbecco's modified Eagle medium (DMEM; Hyclone, SH30243.01, USA) supplemented with 10% fetal bovine serum (FBS; Hyclone, SH30919.03, USA) and interferon-γ (50 U/mL; PeproTech EC Ltd., 300–02, UK) under permissive conditions (33 °C, 10% CO₂). Cells were seeded in six-well plates at 2.0 × 10^5^ cells per well and transfected with EGFP-encoding mRNA (EGFP–mRNA) at 1 μg/mL using Lipofectamine 2000 (ThermoFisher, 11668019, USA), and the medium was replaced with fresh culture medium 3 h after transfection. Total RNA and protein were extracted using the RNeasy Mini Kit (Qiagen, 74104, Germany) and RIPA buffer (Elpis Biotech, EBA-1149, Republic of Korea), respectively. Cell viability was assessed 30 h after transfection using the MTT assay (3-(4,5-dimethylthiazol-2-yl)-2,5-diphenyltetrazolium bromide; Sigma, 1.11714, USA). Lipofectamine and LNP were compared at a matched mRNA dose of 1 μg/mL. Flow cytometry and fluorescence imaging were performed 48 h after treatment. For the LNP condition designated “LNP-3, medium changed at 4 h”, the transfection medium was replaced with fresh culture medium 4 h after treatment; the medium was not exchanged in the other LNP condition. The comparator group received the corresponding delivery vehicle without mRNA under otherwise identical culture and handling conditions and is designated throughout as the vehicle-treated control; MTT values were normalized to this group, defined as 100%. PBS was used as the vehicle rather than a cargo-free LNP because, in this formulation, the particle assembles around the nucleic acid, so a particle of equivalent composition and physical properties cannot be produced in the absence of mRNA; PBS is therefore the appropriate control for the delivery buffer and the applied volume, consistent with the *in vivo* vehicle-injected (PBS) control described in Section 2.12. All *in vitro* experiments, including cell viability assays, flow cytometry, RT-PCR, and Western blot analysis, were performed in three independent biological replicates (*n* = 3). For the MTT assay, MTT solution was added to each well to a final concentration of 0.5 mg/mL and the plates were returned to the incubator for 4 h; the medium was then removed, the resulting formazan crystals were dissolved in dimethyl sulfoxide, and absorbance was measured at 570 nm with a reference wavelength of 630 nm. For the fluorescence images in Fig. 3A, nuclei were identified from the DAPI signal to give the number of cells per field, and reporter expression is reported as the fraction of DAPI-positive nuclei with an associated EGFP signal in the same field.

### 2.6 Reverse-transcription polymerase chain reaction (RT-PCR)

Following transfection, HEI-OC1 cells were collected by gentle scraping, and total RNA was isolated using the RNeasy® Micro Kit according to the manufacturer's instructions (Qiagen, 74004, Germany). Complementary DNA (cDNA) was synthesized from the purified RNA with the High-Capacity cDNA Reverse Transcription Kit (Applied Biosystems, 4368814, USA). The expression levels of EGFP transcripts were examined using end-point PCR, each performed with 10 ng of cDNA as a template. The specific primers used for EGFP amplification were: forward, 5′-CAT GGT CCT GCT GGA GTT CGT G-3′; and reverse, 5′-CGT CCA TGC CGA GAG TG-3′.

### 2.7 Western blot

HEI-OC1 cells were rinsed thoroughly with ice-cold PBS and collected using a cell scraper. The harvested cells were lysed in 100 μL of RIPA buffer (Elpis Biotech, EBA-1149, Republic of Korea) supplemented with a protease inhibitor cocktail (GenDEPOT, P3100–001, USA). The lysates were centrifuged at 13,000 rpm for 30 min at 4 °C, and the resulting supernatants were transferred to pre-chilled tubes. Equal amounts of protein were resolved by SDS–polyacrylamide gel electrophoresis and subsequently transferred onto nitrocellulose membranes. Membranes were blocked for 1 h in TBS-T (20 mM Tris–HCl, pH 7.6; 137 mM NaCl; 0.01% Tween-20) containing 5% skim milk, followed by overnight incubation at 4 °C with primary antibodies against GFP (rabbit, 1:1000; Cell Signaling Technology, 2555, USA) and β-actin (1:1000; Abcam, ab20272, UK). After three 5-min washes in TBS-T, membranes were incubated with HRP-conjugated goat anti-rabbit IgG (1:1000; Cell Signaling Technology, 7074, USA) for 1 h at room temperature. Protein bands were visualized using an enhanced chemiluminescence detection reagent with ProSieve® QuadColor® Protein Marker (Lonza, 00193837, Switzerland). Western blot analyses were performed using three independent biological replicates (*n* = 3).

### 2.8 Organotypic cultures of postnatal organ of Corti

Cochleae were dissected under sterile conditions. Organotypic explant cultures were prepared from the cochleae of 3-day-old postnatal C57BL/6 mice. The mouse heads were bisected along the mid-sagittal plane, and the cochleae were isolated and transferred into ice-cold Hank's balanced salt solution (HBSS; Welgene, LB003–02, Republic of Korea). The lateral wall tissues (stria vascularis and spiral ligament), the auditory nerve bundle, and spiral ganglion neurons were carefully dissected, preserving the organ of Corti. The cochlear explants were placed onto a culture dish (4-well plate; SPL Life Sciences, Republic of Korea) previously coated with 0.1% porcine skin gelatin (Sigma-Aldrich, G1890, USA). The explants were incubated at 37 °C in a 10% CO_2_ atmosphere in a medium containing 175 μL of high-glucose Dulbecco's modified Eagle's medium (DMEM; Cytiva, SH30081.01, USA) supplemented with 10% FBS (Hyclone, SH30919.03, USA) and 100 μg/mL ampicillin. After 5 h, an additional 325 μL of culture medium was added to submerge the explants. The tissue was allowed to adhere to the gelatin-coated surface under incubator conditions for 24 h. Prior to cochlear dissection, P3 mice were euthanized by carbon dioxide (CO₂) inhalation in accordance with institutional animal welfare guidelines.

### 2.9 LNP treatment of organ culture explants

The cultured specimens were divided into three groups: untreated control (control), non-sucrose mCherry mRNA–LNP-treated (LNP-4), and 5% sucrose mCherry mRNA–LNP-treated (LNP-4 with sucrose). Two cochleae from each mouse were assigned to different groups to minimize individual variation, resulting in 18 cochlear explants per experimental group. After incubating for 24 h, explants in the non-sucrose and 5% sucrose groups were treated with 1 μg/mL of LNP-4 and LNP-4 with sucrose, respectively, both diluted in culture medium. After 4 h, the medium containing LNPs was replaced with a fresh culture medium. All treated and untreated explants were divided into six subgroups, with three explants per subgroup. The explants were then incubated for six different time points: 4 h, 12 h, 24 h, 48 h, 72 h, and 5 days. At each time point, the explants underwent immunofluorescence analysis.

### 2.10 Wholemount immunofluorescence staining

Following fixation with 4% paraformaldehyde (PFA), the tissues were permeabilized using 0.1% Triton X-100 in 1× PBS at room temperature (RT) for 30 min. Blocking was performed using 5% normal goat serum (NGS) in 0.1% Triton X-100 in 1× PBS at RT for 1 h. The cochlear tissues were then incubated with a rabbit polyclonal antibody against mCherry (Invitrogen, PA5–34974, USA) diluted 1:1000 in a blocking solution at 4 °C overnight. The following day, the tissues were washed three times for 5 min each with 1× PBS and incubated with secondary Alexa Fluor 555-conjugated goat anti-rabbit IgG (1:1000; Invitrogen, A21428, USA) at RT for 1 h. Alexa Fluor 488 phalloidin (1:1000; Invitrogen, A12379, USA) was included in the secondary antibody mixture for counterstaining. After washing three times with 1× PBS for 5 min each, the tissues were mounted on glass slides using Fluoromount (Sigma-Aldrich, F4680, USA). The mounted specimens were imaged using a laser scanning confocal microscope (Carl Zeiss, LSM 800, Germany). Three-dimensional reconstruction from the optical sections (*Z*-stacks) was performed using LSM ZEN 2 blue edition software.

### 2.11 Frozen section immunofluorescence staining

The hemidissected mouse heads were processed by removing the skin and brain, isolating the inner ear skull. These specimens were fixed in 4% PFA at 4 °C overnight, then dehydrated in 30% sucrose in 1× PBS at 4 °C overnight. The samples were incubated in a 1:1 mixture of 30% sucrose and O.C.T. compound (Leica, 3801480, Germany) at room temperature, embedded in cryomolds and frozen on dry ice. Frozen blocks were sectioned at a thickness of 12 μm. For immunostaining, sections were outlined with a hydrophobic pen and washed with 1× PBS. Tissue permeabilization was performed using 0.1% Triton X-100 for 30 min, followed by antigen blocking with 3% goat serum and 2% BSA for 1 h. Primary antibody incubation utilized mCherry rabbit polyclonal antibody (1:1000; Invitrogen, PA5–34974, USA) at 4 °C overnight. The following day, tissues were washed five times with 1× PBS, each for 5 min. A secondary antibody (1:1000; Alexa Fluor 555-conjugated goat anti-rabbit IgG, Invitrogen, A21428, USA) was applied, and the staining process continued as described. Alexa Fluor 488 phalloidin (Invitrogen, A12379, USA) was utilized for counterstaining at a dilution of 1:1000 and incubated for 1 h. Following this, tissues underwent five washes with 1× PBS and were subsequently mounted using Fluoromount (Sigma-Aldrich, F4680, USA). Image analysis was conducted using a laser scanning confocal microscope (Carl Zeiss, LSM 800, Germany) in conjunction with LSM ZEN 2 blue edition software.

### 2.12 Round window membrane injections

Neonatal mice (P0–P2) were anesthetized by gradual induced hypothermia, the accepted method for rodent neonates of this age and recognized for pups up to seven days old, in preference to injectable agents whose pharmacokinetics and safety margins at this stage are poorly characterized. Pups were placed on a silicone pad overlying ice so that they were never in direct contact with the ice, allowing body temperature to fall gradually. Induction did not exceed 2–3 min, and adequate depth of anesthesia was confirmed by the absence of a response to toe pinch before the incision was made. A skin incision was made on the posterior aspect of the left ear using microsurgical scissors. After careful muscle dissection, the Y-vessel and tympanic ring were located, followed by identifying the round window above the stapedial artery. A glass micropipette was filled with either 1 μL of LNPs (46 ng) or LNP combined with 5% sucrose (35 ng) and was inserted into the RWM. The injection was administered at a rate of 8 nL/s using a Nanoliter 2020 (WPI, USA). Control animals underwent the identical surgical approach and received 1 μL of PBS at the same injection rate, and are designated the vehicle-injected control. An LNP without cargo was not used as the vehicle because, in this formulation, the particle assembles around the nucleic acid, so a particle of equivalent composition and physical properties cannot be produced in the absence of mRNA; PBS is therefore the appropriate control for the surgical procedure and the injected volume. On completion of the procedure, pups were rewarmed gradually at room temperature and then on a heating pad until normal color, spontaneous movement and body temperature had returned, after which they were returned to the dam and maternal acceptance was confirmed by direct observation. No systemic analgesic was administered; this was specified in and approved as part of IACUC protocol No. 2022–0147, on the recorded grounds that the procedure is brief and minimally invasive and that the pharmacokinetics, efficacy and safety margins of systemic analgesics in P0–P2 pups are poorly characterized. At the experimental endpoint, animals were euthanized by carbon dioxide (CO₂) inhalation. Round window injections were performed in seven animals per group (*n* = 7).

### 2.13 Auditory brainstem response (ABR)

All experiments were conducted in a soundproof environment to evaluate auditory function, and ABR recordings were obtained using an ABR workstation (RZ6, Tucker Davis Technologies, Inc. (TDT), USA). Prior to ABR testing, mice were anesthetized *via* intraperitoneal injection of a mixture containing Alfaxalone (4 mg/100 g) and xylazine hydrochloride (0.13 mg/100 g). The animals were placed on a heating pad throughout the procedure to maintain a body temperature of 37 °C. The positive electrode was inserted subcutaneously into the cortex, while the negative electrode was positioned at the ipsilateral mastoid and the ground electrode at the contralateral mastoid. Neural signals were amplified 20-fold using a preamplifier (RA4PA Medusa preamp, TDT, USA) connected to a headstage (RA4LI, TDT, USA) with a band-pass filter set between 300 Hz and 3 kHz. Auditory stimuli included click sounds and tone bursts at frequencies of 4, 8, 16, and 32 kHz, generated using SigGenRP and RP2.1 real-time processors in combination with a programmable attenuator (PA5, TDT, USA) and delivered *via* a speaker driver (ED1, TDT, USA) and a magnetic speaker (EC1, TDT, USA). Sound stimuli were initially presented at 90 dB and gradually decreased in 5 dB increments until the hearing threshold was established, with each stimulus repeated 300 times. Testing was performed at postnatal day 20 and at four weeks in *n* = 7 animals per group. In addition to threshold and click-evoked wave I amplitude, wave I latency was measured from the stored waveform records at each stimulus level and is reported as a latency–intensity function.

### 2.14 Experimental design

This study was designed to optimize an mRNA-loaded LNP formulation for inner ear gene delivery and to evaluate its transfection efficiency and biodistribution through sequential *in vitro*, *ex vivo*, and *in vivo* experiments. The overall study consisted of four consecutive phases.

In the first phase, a DoE approach employing a 3^2^ full factorial design was used to optimize the LNP formulation. The aqueous-to-ethanol ratio and the N/P ratio were defined as CPPs, whereas particle size, PDI, and EE were selected as CQAs. Each formulation generated by the factorial design was evaluated independently.

In the second phase, the optimized LNP formulation was characterized and compared with alternative formulations containing different ionizable lipids or produced using different manufacturing processes. Physicochemical properties, including particle size, PDI, encapsulation efficiency, and storage stability, were evaluated.

In the third phase, *in vitro* transfection experiments were performed using HEI-OC1 auditory cells. Experimental groups consisted of vehicle-treated control cells, Lipofectamine-mediated mRNA delivery, small-scale manufactured LNPs, and microfluidic-manufactured LNPs where appropriate. The experimental unit for all *in vitro* experiments was a single cell culture well, and all experiments were performed using three independent biological replicates (*n* = 3). Primary outcome measures included transfection efficiency, EGFP expression, protein expression, and cell viability.

In the fourth phase, the biodistribution of mRNA-loaded LNPs was investigated using organotypic cochlear explant cultures and neonatal mouse models. For organotypic cultures, untreated explants served as controls and were compared with explants treated with standard LNPs or sucrose-containing LNP formulations. The experimental unit was a single cochlear explant, and three independent cochlear explants (*n* = 3) were analyzed for each treatment group.

For the *in vivo* experiments, neonatal C57BL/6 mice received mRNA-loaded LNPs either through RWM administration or scala media injection to compare route-dependent gene delivery. Vehicle-injected (PBS) control animals, which underwent the identical surgical approach, served as controls for auditory function and tissue morphology where appropriate. The experimental unit for all *in vivo* experiments was a single mouse. Seven animals (*n* = 7) were included in each experimental group unless otherwise specified. ABR measurements, fluorescence imaging, and histological analyses were performed to evaluate hearing preservation and biodistribution following treatment.

The study was designed prospectively before initiation of the experiments. All experimental procedures, imaging conditions, and analytical methods were standardized across experimental groups to minimize technical variation. Comparisons between groups were predefined before data collection, and all outcome measures were analyzed according to the statistical methods described below.

### 2.15 Sample size determination

Sample sizes were determined based on previous studies using comparable *in vitro*, *ex vivo*, and neonatal mouse models for inner ear gene delivery, together with practical and ethical considerations in accordance with the principles of the 3Rs (Replacement, Reduction, and Refinement). No formal *a priori* statistical power calculation was performed because the primary objective of this study was to optimize the mRNA-loaded LNP formulation and to evaluate its transfection efficiency and biodistribution rather than to detect a predefined treatment effect.

For all *in vitro* experiments, three independent biological replicates (*n* = 3) were performed for each experimental condition, with each biological replicate consisting of a single cell culture well. Cell viability assays, RT-PCR, Western blot analyses, flow cytometry, and fluorescence imaging were conducted using these independent biological replicates.

For organotypic cochlear explant experiments, three independent cochlear explants (*n* = 3) were analyzed for each treatment condition at each experimental time point unless otherwise indicated. Each cochlear explant was considered one experimental unit. A total of 15 postnatal day-3 (P3) mice were used to prepare the cochlear explant cultures included in this study.

For *in vivo* biodistribution studies, seven neonatal mice (*n* = 7) were included in each experimental group unless otherwise specified in the corresponding figure legends. Each mouse was regarded as one experimental unit. Sample sizes were selected because they were sufficient to evaluate the reproducibility of biodistribution patterns, transgene expression, and hearing preservation while minimizing the number of animals used. The selected sample sizes were consistent with those commonly used in previous exploratory studies of inner ear gene delivery and were considered sufficient to demonstrate reproducible biological outcomes while avoiding unnecessary animal use.

Seven neonatal mice per experimental group (*n* = 7) were used for the *in vivo* studies, which comprised RWM injection, scala media injection, ABR assessment, and tissue collection for histological and immunofluorescence analyses.

### 2.16 Inclusion and exclusion criteria

Inclusion and exclusion criteria were established prior to the initiation of the study. Healthy C57BL/6 mice without visible congenital abnormalities or signs of illness were included in the *in vivo* experiments. Cochlear explants exhibiting intact cochlear morphology after dissection and successful attachment to the culture surface were included in the *ex vivo* analyses. HEI-OC1 cells showing normal morphology and viability at the time of transfection were included in all *in vitro* experiments.

Experimental units were excluded only when predefined technical criteria were met, including unsuccessful surgical injection, substantial leakage of the injected solution, failure of cochlear explant attachment or tissue integrity during culture, contamination of cell cultures, or technical failure during sample preparation or image acquisition. These exclusion criteria were established before data collection and were unrelated to the experimental outcomes.

No animals, cochlear explants, cell culture samples, or individual data points were excluded from the final analyses based on the observed experimental results. Likewise, no additional experimental units were added after completion of the experiments for the purpose of achieving statistical significance.

The exact number of experimental units analyzed for each experiment is reported in the corresponding figure legends and Methods section.

### 2.17 Randomization and blinding

Animals were randomly allocated to the experimental groups before treatment using a simple randomization procedure. Following confirmation that animals met the predefined inclusion criteria, each animal was assigned a unique identification number and randomly allocated to the corresponding experimental group. Where possible, littermates were distributed across different experimental groups to minimize potential litter-specific effects.

To minimize potential confounding factors, all animals were housed under identical environmental conditions, including the same temperature, humidity, light/dark cycle, food, and water availability. Surgical procedures, LNP preparation, injections, ABR recordings, tissue collection, and histological analyses were performed according to standardized protocols using identical experimental conditions. Whenever possible, experiments from different treatment groups were performed during the same experimental period to reduce potential batch effects. Sample processing, fluorescence imaging, and image acquisition were conducted using identical microscope settings and acquisition parameters for all experimental groups.

Formal blinding was not implemented during treatment allocation or surgical procedures because investigators were necessarily aware of the administered treatment. However, quantitative image analyses and data processing were performed according to predefined objective criteria using identical analysis parameters for all samples to minimize observer bias. Statistical analyses were conducted using predefined statistical methods without modifying the analysis strategy after inspection of the experimental outcomes.

### 2.18 Outcome measures

Outcome measures were predefined before the initiation of the study according to the objectives of each experimental phase. The primary objective of this study was to establish an optimized mRNA-loaded LNP formulation for inner ear gene delivery and to evaluate its transfection efficiency and biodistribution.

The primary outcome measures were:

(i) optimization of the physicochemical properties of mRNA-loaded LNPs, including particle size, PDI, and EE, determined using the DoE approach;
(ii) evaluation of mRNA transfection efficiency in HEI-OC1 auditory cells by flow cytometry, fluorescence microscopy, RT-PCR, and Western blot analysis; and.
(iii) assessment of the biodistribution of mRNA-loaded LNPs within the inner ear following RWM or scala media administration using immunofluorescence imaging.

The secondary outcome measures included comparison of transfection efficiency between Lipofectamine- and LNP-mediated mRNA delivery systems, cell viability after transfection, physicochemical stability of LNP formulations during storage, ABR thresholds and click-evoked wave I amplitudes following neonatal injection, and qualitative evaluation of cochlear morphology and transgene expression in organotypic cochlear explants and cochlear tissue sections.

All outcome measures were predefined before data collection and were analyzed according to the statistical methods described in Section 2.19. The primary outcome measures were selected because they directly reflected the objectives of optimizing the LNP formulation and evaluating the efficiency and distribution of mRNA delivery within the inner ear.

### 2.19 Statistical analysis

Statistical analyses were performed using GraphPad Prism version 10 (GraphPad Software, USA). Unless otherwise indicated, all *in vitro* experiments, including cell viability assays, flow cytometry, RT-PCR, Western blot analysis, and physicochemical characterization of lipid nanoparticles, were independently performed using three biological replicates (*n* = 3). Sample sizes for organotypic cochlear explant cultures and *in vivo* experiments are described in the corresponding Methods sections and figure legends.

Data are presented as the mean ± standard error of the mean (SEM). Prior to statistical analyses, data were assessed for normality using the Shapiro–Wilk test. Homogeneity of variance was evaluated using the Brown–Forsythe test where appropriate. Parametric statistical analyses were performed only when these assumptions were satisfied. If the assumptions for parametric analysis were not met, appropriate non-parametric statistical methods were considered. However, all datasets included in the final analyses satisfied the assumptions required for the statistical tests applied.

Comparisons between two groups were performed using an unpaired two-tailed Student's *t*-test where appropriate. Comparisons among multiple groups were performed using one-way analysis of variance (ANOVA) followed by Dunnett's multiple comparisons test. Click-evoked ABR thresholds were compared using an unpaired two-tailed Student's *t*-test. Tone-burst ABR thresholds and click-evoked wave I amplitude and wave I latency–intensity functions were analyzed using two-way repeated-measures ANOVA with Geisser–Greenhouse correction, followed by Bonferroni's multiple comparisons test. For the auditory outcomes, effect sizes are reported as the between-group mean difference with 95% confidence intervals alongside *p* values.

All statistical analyses were predefined before data collection and performed using two-sided tests. A *P* value of <0.05 was considered statistically significant.

## 3 Results and discussion

### 3.1 Design of experiments for preparing an appropriate LNP formulation

Establishing a suitable LNP formulation is imperative to achieve efficient mRNA delivery to the inner ear. Although numerous studies have documented the development and manufacturing of LNPs, these formulations have been prepared under diverse conditions, except for the molar ratio of lipid components (Zhang et al., 2023). Thus, a more systematic process incorporating the identification of CQAs and CPPs and the application of a DoE approach is warranted to establish an appropriate LNP formulation.

The key CQAs associated with LNPs include particle size, PDI, and EE; particle size significantly impacts mRNA encapsulation efficiency and half-life, which is crucial in enhancing cellular uptake, with smaller particles generally yielding higher delivery efficiency (Tenchov et al., 2021; Evers et al., 2018). Notably, the efficiency of gene and drug delivery *via* nanoparticles is often inversely proportional to particle size (Valente et al., 2017), underscoring the necessity of designing LNPs with an appropriate size. Furthermore, PDI reflects the homogeneity of LNP formulations, where higher values indicate greater polydispersity, potentially diminishing gene delivery efficiency (Mehraji and DeVoe, 2024). Elevated EE values also suggest that more mRNA is successfully encapsulated within LNPs, thereby protecting the cargo from enzymatic degradation and ensuring stable gene delivery. Consequently, maximizing EE enhances overall gene delivery efficiency. Based on these considerations, we established target CQAs: particle size ≤100 nm (Tenchov et al., 2021; Evers et al., 2018), PDI ≤0.3 (Mehraji and DeVoe, 2024), and EE ≥80%. Beyond achieving these CQA targets, our objective was to identify a suitable LNP formulation that maximizes EE while minimizing particle size and PDI.

Thus, it is crucial to identify CPPs that significantly impact these attributes to achieve an appropriate LNP formulation that meets the CQA criteria. Considering the mechanism involved in LNP formulation *via* ethanol injection (Maeki et al., 2017), we identified the aqueous solution-to-ethanol ratio (*v*/v) and the nitrogen-to-phosphate ratio (N/P ratio) as key factors. The aqueous-to-ethanol ratio is an important parameter because it influences the local solvent environment during lipid-RNA assembly and thereby affects lipid precipitation, particle formation, and RNA encapsulation. Although a 3:1 aqueous-to-ethanol ratio is frequently used, different ratios within the range of 1:1 to 5:1 have also been reported (Abdelkarim et al., 2025; Chen et al., 2025; Mukherjee et al., 2026), highlighting the need for experimental optimization under formulation conditions. Additionally, the N/P ratio is known to influence RNA EE%, particle size, and subsequent cellular uptake. Given the wide range of N/P ratios reported in the literature (Blakney et al., 2021; Carrasco et al., 2021; Guruge et al., 2025), the N/P ratio was also selected as an independent variable for DoE optimization. To our knowledge, systematic DoE optimization linking both the aqueous-to-ethanol ratio and N/P ratio and simultaneously considering LNP physicochemical properties has not been previously reported. The DoE model provides quantitative evidence of parameter interactions, defines the response behavior within the investigated design space, and enables identification of an optimized formulation condition simultaneously considering multiple CQAs.

Accordingly, a DoE approach using 3^2^ full factorial design was employed, in which these CPPs were designated independent variables, whereas particle size, PDI, and EE% were dependent variables (Table 1). The ionizable lipid, a major component in LNP formulation, was fixed as SM-102 during the design and execution of the experiments. Next, DoE-based 3-dimensional surface and contour plots were generated to examine the relationship between CPPs and CQAs (Fig. 1). Based on the DoE analysis, the quantitative relationships between the independent variables (A: aqueous-to-ethanol ratio [*v*/v] and B: N/P ratio) and the responses, including particle size (Y_1_), PDI (Y_2_), and EE% (Y_3_), were described by the following second-order polynomial equations:

(1) $$Y_{1}=94.057–8.97\text{5A}-9.16\text{0B}+19.33{\text{5A}}^{2}$$

(2) $$Y_{2}=0.114–0.11\text{9A}-0.0243B+0.12{\text{4A}}^{2}$$

(3) $$Y_{3}=91.490+37.75\text{2A}+2.78\text{5B}-45.69{\text{5A}}^{2}$$

**Table 1:** Experimental runs of 3^2^ full factorial designs for LNP preparation.

Run	Independent factors (X)		Response variables (Y)		
	X_1_	X_2_	Y_1_	Y_2_	Y_3_
LN1	5	2	109.3	0.151	72.09
LN2	5	5	104.2	0.117	87.94
LN3	5	8	99.75	0.091	90.61
LN4	1	5	134.6	0.344	N.D.
LN5	1	2	130.2	0.397	15.27
LN6	3	5	90.88	0.108	92.89
LN7	3	8	86.89	0.108	93.09
LN8	1	8	102.3	0.33	8.86
LN9	3	2	104.4	0.127	88.49

X_1_: aqueous-to-ethanol ratio (*v*/v), X_2_: N/P ratio, Y_1_: Particle size, Y_2_: PDI, Y_3_: EE (%), N.D.: Not detected.

**Fig. 1:**
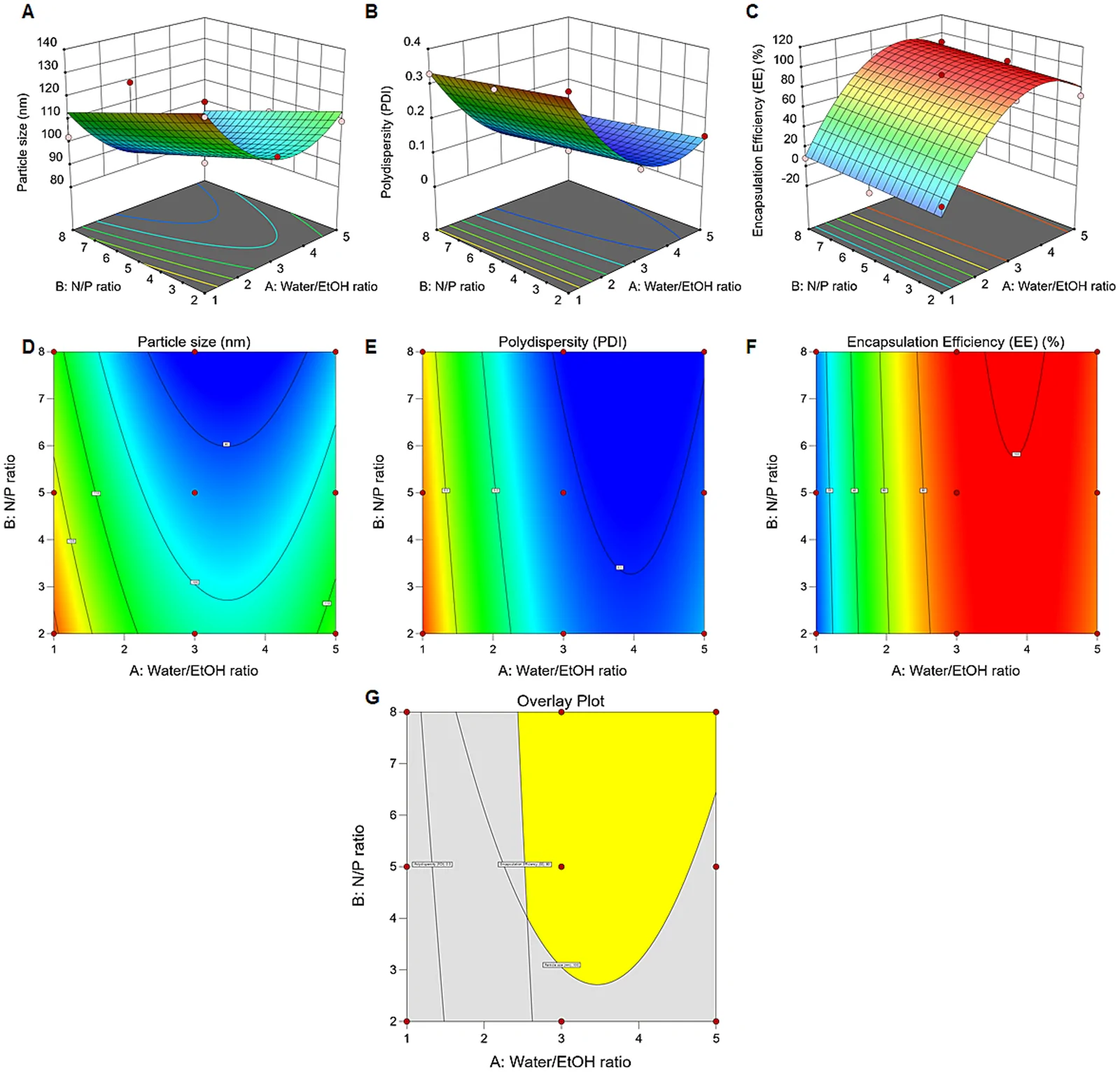
Three-dimensional surface plots, contour plots, and overlay plots based on the design of experiments (DoE). (A–C) Three-dimensional surface plots of LNP physicochemical properties – particle size (A), PDI (B), and EE (C) – as the function of aqueous-to-ethanol ratio and N/P ratio. (D–F) Contour plots derived from the three-dimensional surface plot. (G) Overlay plot based on CQAs to determine suitable conditions for LNP formulation.

As shown in Tables S1–S6 and Fig. S1, the fitted models were statistically significant (*p* < 0.05). In addition, the normal probability plots of the residuals indicated that the residuals were approximately normally distributed, and the actual-*versus*-predicted plots demonstrated good agreement between the experimentally observed and model-predicted values (Fig. S1). These results support the adequacy of the fitted models for describing the experimental responses.

Based on the model equations, all three responses were associated with the investigated formulation parameters, with the quadratic effect of factor A (A^2^) showing a particularly pronounced contribution to the responses. These results suggest that although the N/P ratio also affected the CQAs, the aqueous-to-ethanol ratio had a greater influence on the physicochemical characteristics of the LNPs under the conditions investigated. The DoE-derived design space further indicated that an aqueous-to-ethanol ratio of approximately 3 for an N/P ratio of ≥5 resulted in LNPs meeting the predefined target criteria for particle size, PDI, and EE%.

Moreover, the three CQAs were closely associated with the functional EGFP-encoded mRNA delivery performance of the LNP formulations. To examine whether the CQA-based selection was consistent with biological mRNA delivery performance before selecting the final formulation, the LNPs prepared during the experimental runs were additionally evaluated for transfection efficiency in NIH/3T3 cells, a commonly used reference cell model for assessing gene delivery (Ren et al., 2022). This experiment was conducted as a preliminary ranking assessment across the nine factorial formulations using a robust cell model. Importantly, this assessment was not intended to establish cochlear tropism or predict inner ear cell-specific cell delivery. The selected formulation was subsequently evaluated in the auditory-relevant HEI-OC1 cell model, followed by assessment in cochlear explants and *in vivo* biodistribution and mRNA expression.

Although EGFP expression translated from the delivered mRNA was not included as a formal response in the primary DoE optimization, we additionally evaluated the biological transfection performance of the nine LNP formulations in NIH/3T3 cells (Table S7, Fig. S2). GFP fluorescence intensity was used as a response to preliminarily assess the transfection performance of the LNP formulations. A separate response surface analysis of EGFP expression demonstrated a statistically significant relationship between the investigated formulation factors and GFP expression (Fig. S2). Importantly, the region associated with higher GFP expression substantially overlapped with the design space identified based on particle size, PDI, and EE% (Fig. 1G, Fig. S2A, B). These findings provide additional biological support for the formulation region selected through the physicochemical DoE.

The corresponding response surface and contour plots, together with the ANOVA and model fit statistics, are provided in Tables S8 and S9. The EGFP expression model was statistically significant, and the normal probability plot of the residuals and the actual-*versus*-predicted plot for the natural log (ln)-transformed EGFP expression further supported the adequacy of the fitted model (Fig. S2C, D). The regression equation for EGFP expression (Y_4_) was as follows:

(4) $$\ln\;\left(Y_{4}\right)=10.034+1.28\text{0A}+0.61\text{7B}-0.44\text{2AB}-1.35{\text{2A}}^{2}$$

Comparison of green fluorescence following transfection with EGFP–mRNA revealed that the highest gene expression was achieved at an N/P ratio of 8 and an aqueous-to-ethanol ratio of 3 (Table S7). Importantly, this formulation condition simultaneously satisfied the predefined CQA criteria, including desired particle size, PDI and EE%, and was located within the DoE-derived design space (Fig. 1G). Consequently, this LNP formulation was selected as the suitable condition for subsequent evaluation of mRNA delivery.

### 3.2 Establishment of scale-up production process of LNPs

In addition to establishing the appropriate LNP manufacturing process, selecting an appropriate ionizable lipid is critical for effective gene delivery. Among the ionizable lipids approved for clinical use in mRNA-based vaccines and therapeutics, SM-102 and ALC-0315 have been extensively utilized, particularly in COVID-19 vaccines (Zhang et al., 2023).

To ascertain which ionizable lipid is most effective for mRNA delivery, we compared the particle size, EE, and PDI of LNPs formulated under the identical process conditions (N/P ratio = 8; aqueous-to-ethanol ratio = 3) while varying only the ionizable lipid component (Table S10). Comparative analysis of the size distribution of LNPs formulated with different ionizable lipids revealed that LNPs based on SM-102 (LN7 in Table 1; LNP-1 in Table S10) exhibited an average particle size below 100 nm. In contrast, those formulated with ALC-0315 (LNP-2 in Table S10) formed larger particle sizes, averaging above 100 nm. Additionally, using ALC-0315 resulted in a slightly lower EE (<70%), consistent with previous studies comparing the delivery efficiency of these two ionizable lipids (Zhang et al., 2023). Consequently, LNPs formulated with ALC-0315 did not meet the predefined target CQA. Based on these findings, SM-102 was selected as the preferred ionizable lipid for mRNA delivery to the inner ear.

Establishing a scalable manufacturing process for LNP production is essential alongside selecting an appropriate ionizable lipid. A simple large-scale bulk formulation approach tends to increase particle size and reduce EE, which can significantly impact transfection efficiency. Therefore, such methods are unsuitable for producing nanoparticles intended for preclinical and clinical applications (Shepherd et al., 2021). To address this challenge, we applied the appropriate LNP formulation to a continuous-flow microfluidic system for large-scale production and compared the characteristics of the LNPs produced using this method with those synthesized on a small scale (Table S10). The results demonstrated that LNPs manufactured using the microfluidic system (LNP-3 in Table S10) exhibited compact small particle sizes (mean size: 40–60 nm), low PDI (<0.15), and high EE (>90%), consistent with those produced on a small scale, while also meeting the predefined CQA criteria. In conclusion, we successfully established an appropriate manufacturing process based on DoE and developed a scalable production system utilizing a microfluidic device.

Additionally, we investigated whether the suitable LNP formulation could be effectively applied to mRNAs encoding proteins other than EGFP to enable fluorescence-based tracking of LNP biodistribution *in vivo*. Notably, despite GFP being a stable fluorescent protein that enables non-invasive monitoring of LNP-mediated mRNA delivery in live cells, limitations have been noted within *in vivo* environments due to tissue autofluorescence, which hinders effective monitoring (Lifante et al., 2020). Therefore, we encapsulated mCherry-encoding mRNA (mCherry–mRNA), which expresses a red-shifted fluorescent protein instead of GFP, into LNPs to facilitate *in vivo* monitoring and evaluated the physicochemical properties (Kim et al., 2020). The results demonstrated that despite differences in mRNA sequences, the mCherry–mRNA-loaded LNPs (LNP-4) exhibited CQAs comparable to those of EGFP–mRNA-loaded LNPs, including size, PDI, and EE (Fig. S3; Table S11). Moreover, to enable long-term storage under low-temperature conditions (< −20 °C) for *in vivo* experiments, we evaluated an LNP formulation prepared in a solution with 5% sucrose contained as a cryoprotectant. The results indicated that the particle size, PDI, and EE remained stable without significant changes, satisfying the CQA (Fig. S4; Table S11). Utilizing this LNP platform, we monitored *in vitro* distribution using EGFP–mRNA-loaded LNPs, while *in vivo* LNP distribution was assessed using mCherry–mRNA-loaded LNPs.

### 3.3 Evaluation of gene delivery distribution in an auditory cell line

To assess the efficacy of the LNPs in delivering genes to auditory inner ear cells, we treated HEI-OC1 cells with LNPs under *in vitro* conditions and analyzed the distribution of transfected cells using flow cytometry. Additionally, to compare gene delivery efficiency, we conducted a distribution analysis using Lipofectamine, which is regarded as the gold standard for transfection reagents (Fig. 2) (Cardarelli et al., 2016). Furthermore, we performed a cell viability assay using the same mRNA concentration to evaluate potential cytotoxicity (Fig. 3C). The results indicated that while less than 50% of cells were successfully transfected using Lipofectamine, successful gene delivery occurred in over 80% of cells using LNPs. Moreover, LNPs synthesized using both the small-scale process (LNP-1) and the large-scale microfluidic device (LNP-3) exhibited similar gene delivery efficiencies. Additionally, the Lipofectamine-treated group demonstrated cytotoxic effects at the tested mRNA concentration, whereas the LNPs exhibited minimal toxicity.

**Fig. 2:**
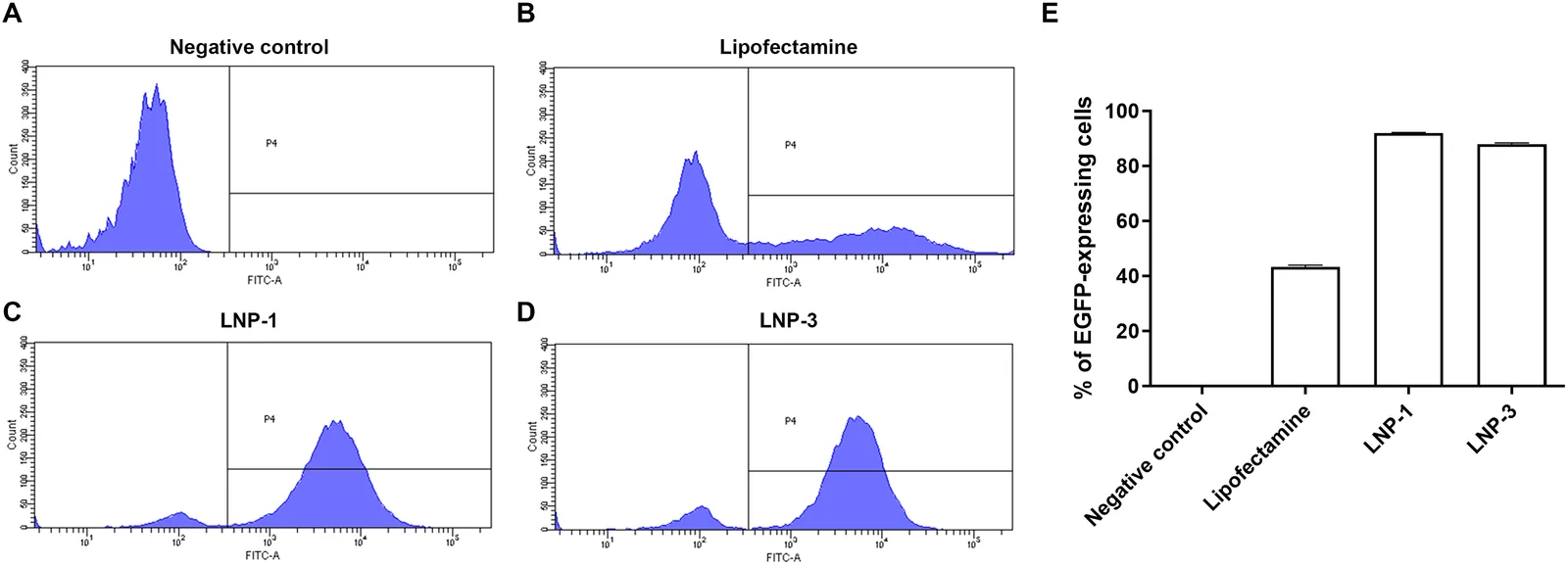
Flow cytometry assay of gene transfection distribution. (A) Flow cytometry analysis of the negative control group (vehicle-treated). (B) Flow cytometry analysis of the Lipofectamine-treated group. (C) Flow cytometry analysis of LNP-treated manufactured on a small scale (LNP-1). (D) Flow cytometry analysis of LNP-treated group manufactured under microfluidic device (LNP-3). (E) Comparison of EGFP expressing cells based on flow cytometry analysis.

**Fig. 3:**
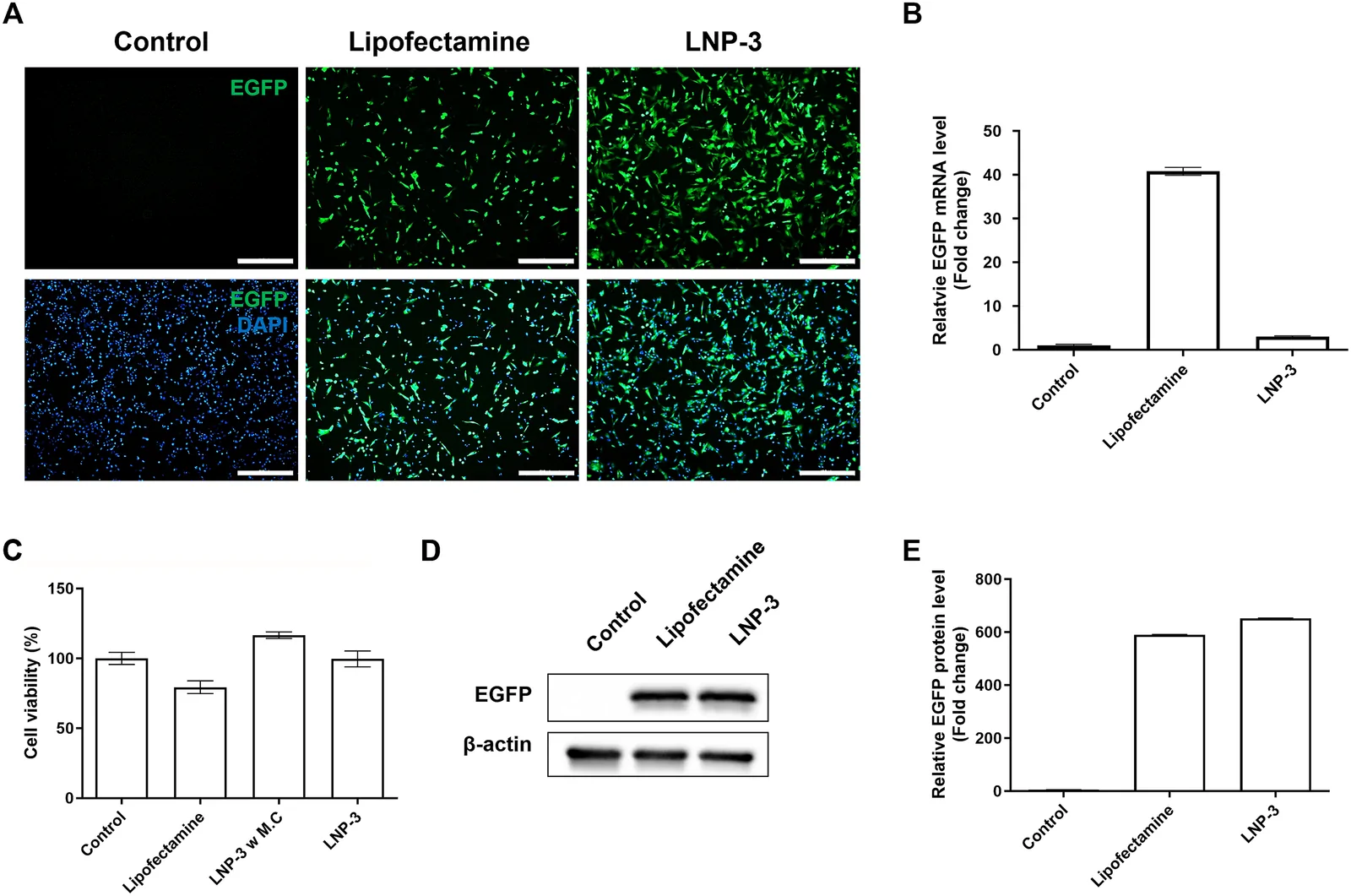
*In vitro* experiments to confirm gene transfection efficacy and mRNA delivery quantity and expression. (A) Live cell image after treatment of Lipofectamine–mRNA complex and LNP. (B) Quantification of mRNA levels after gene transfection. (C) Cell viability test against HEI-OC1 cell. (D–E) Western blot experiment of the EGFP expression. Scale bars: 400 μm.

To further validate these findings, we compared GFP gene expression using live-cell imaging, examined nuclear-stained cells *via* fluorescence microscopy, and quantified EGFP expression using Western blot analysis (Fig. 3). Live-cell imaging revealed that EGFP–mRNA delivered *via* Lipofectamine resulted in strong green fluorescence in only a subset of cells, with some cells exhibiting no fluorescence. In contrast, LNP-mediated EGFP–mRNA delivery demonstrated uniform green fluorescence across the entire cell population (Fig. 3A). We previously described this in terms of co-localization between the EGFP and DAPI signals. That description was inaccurate: DAPI labels nuclei, whereas EGFP is distributed in the cytoplasm and nucleus depending on the construct, so the two are not expected to colocalize. What the images show is the proportion of DAPI-positive nuclei with an associated EGFP signal in the same field—that is, the fraction of cells expressing the reporter. Quantified in this way, the fraction of nuclei with an associated cytoplasmic EGFP signal was higher and more evenly distributed after LNP treatment than after Lipofectamine treatment (Fig. 3A). Previous studies on gene transfection using cationic agents such as Lipofectamine and PEI have reported that gene delivery often occurs localized rather than evenly distributed across all cells. In these non-viral agents, achieving uniform transfection typically necessitates introducing or modifying additional enhancers (Roise et al., 2022; Algarra and Gonzalez-Munoz, 2024). Conversely, the LNP-mediated mRNA delivery system demonstrated increased transfection uniformity, even without specific enhancers.

The three assays used here measure different things, and this accounts for what might otherwise appear to be a discrepancy between them. Flow cytometry reports the proportion of cells above a positivity threshold; Western blotting reports total protein integrated across the pooled population; and bulk RT-PCR reports transcript associated with the harvested cells, including transcript that remains surface-bound or endosomally retained and never reaches the cytosol, so that it measures association rather than functional delivery. Lipofectamine produced strong EGFP expression concentrated in a relatively small number of cells, whereas the LNP produced more moderate expression distributed across a much larger fraction of the population; high expression in few cells and moderate expression in many cells integrate to a comparable pooled protein signal even when the number of expressing cells differs substantially. Although transcript levels were higher with Lipofectamine (Fig. 3B) and overall protein expression remained comparable, the LNP demonstrated an advantage in population-wide delivery uniformity. While the Lipofectamine-based gene delivery system resulted in localized EGFP expression in a subset of cells, the LNP-based system enabled uniform EGFP expression across the entire cell population (Fig. 2, Fig. 3A). Furthermore, cell viability was higher in both conditions where LNPs were applied, regardless of whether a media change was performed or not (Fig. 3C), compared to Lipofectamine treatment. Normalized viability values for the LNP condition in which medium was changed at 4 h lie marginally above 100%. Because MTT values are normalized to the vehicle-treated control defined as 100%, that control carries no variance in the normalized plot while treated groups do, and values modestly above 100% fall within the expected dispersion of the assay; this group did not differ significantly from 100% by one-sample *t*-test. The most likely contributing factor is the medium change itself, which supplies fresh nutrients and can modestly increase metabolic activity relative to wells that were not refreshed. We present this as a plausible explanation rather than a demonstrated one, and the conclusion drawn is limited to the observation that no reduction in metabolic viability was detected under the LNP conditions tested. Additionally, Western blot analysis revealed that overall protein expression levels were similar between the two groups (Fig. 3D and E). This suggests that the inhomogeneous gene transfection observed using Lipofectamine could lead to inconsistencies in gene expression. Such inhomogeneous distribution may further diminish gene delivery efficiency *in vivo*, ultimately limiting the therapeutic efficacy of mRNA-based treatments (Gyorgy et al., 2017). The supported conclusion is therefore narrow: at a matched mRNA dose, the LNP formulation produced reporter expression in a larger and more uniformly distributed fraction of HEI-OC1 cells than Lipofectamine, with comparable total reporter protein and without a detectable reduction in viability. HEI-OC1 is a conditionally immortalized monolayer line and lacks the junctional barriers, fluid compartmentalization and cell-type diversity of the intact cochlea; as shown below, those features rather than intrinsic cellular permissiveness substantially affect delivery *in vivo*. Accordingly, the monolayer transfection results are interpreted as evidence of cellular transfection performance rather than as a direct indicator of cochlear targeting *in vivo*.

### 3.4 Route-dependent gene transduction in the inner ear: differential targeting *via* round window membrane and scala media administration

The route of administration significantly influences the efficiency and spatial distribution of gene delivery within the inner ear. This study evaluated the transduction patterns of mCherry–mRNA LNPs following two distinct delivery approaches: RWM administration and direct scala media injection. Our findings highlight the differential transduction profiles achieved *via* these routes and underscore the anatomical and physiological barriers that limit gene transfer to hair cells.

RWM administration is a well-established method for delivering genetic cargo to the inner ear, facilitating the broad distribution of nanoparticles throughout the perilymphatic compartment. To confirm the procedural and LNP safety of this approach, auditory function tests conducted at postnatal day 20 (P20), a stage when hearing ability is established. All injected mice exhibited normal hearing. Auditory brainstem response measurements revealed no differences compared with vehicle-injected (PBS) control mice in click and tone-burst thresholds, click-evoked wave I amplitudes, or wave I latency–intensity functions (Fig. 4A–D; *n* = 7 per group), indicating that the injection procedure did not induce detectable auditory damage. Although the treated groups showed numerically favorable values across several indicators, none of these differences reached statistical significance, and in a cohort of this size such consistent numerical ordering can arise by chance; we do not attribute it to a treatment effect, and no mechanism by which LNP administration would improve thresholds beyond normal is supported by these data. Fig. 4 is presented as a procedural tolerability assessment rather than as a formally powered equivalence study, and small effects below the resolution of this cohort cannot be excluded.

**Fig. 4:**
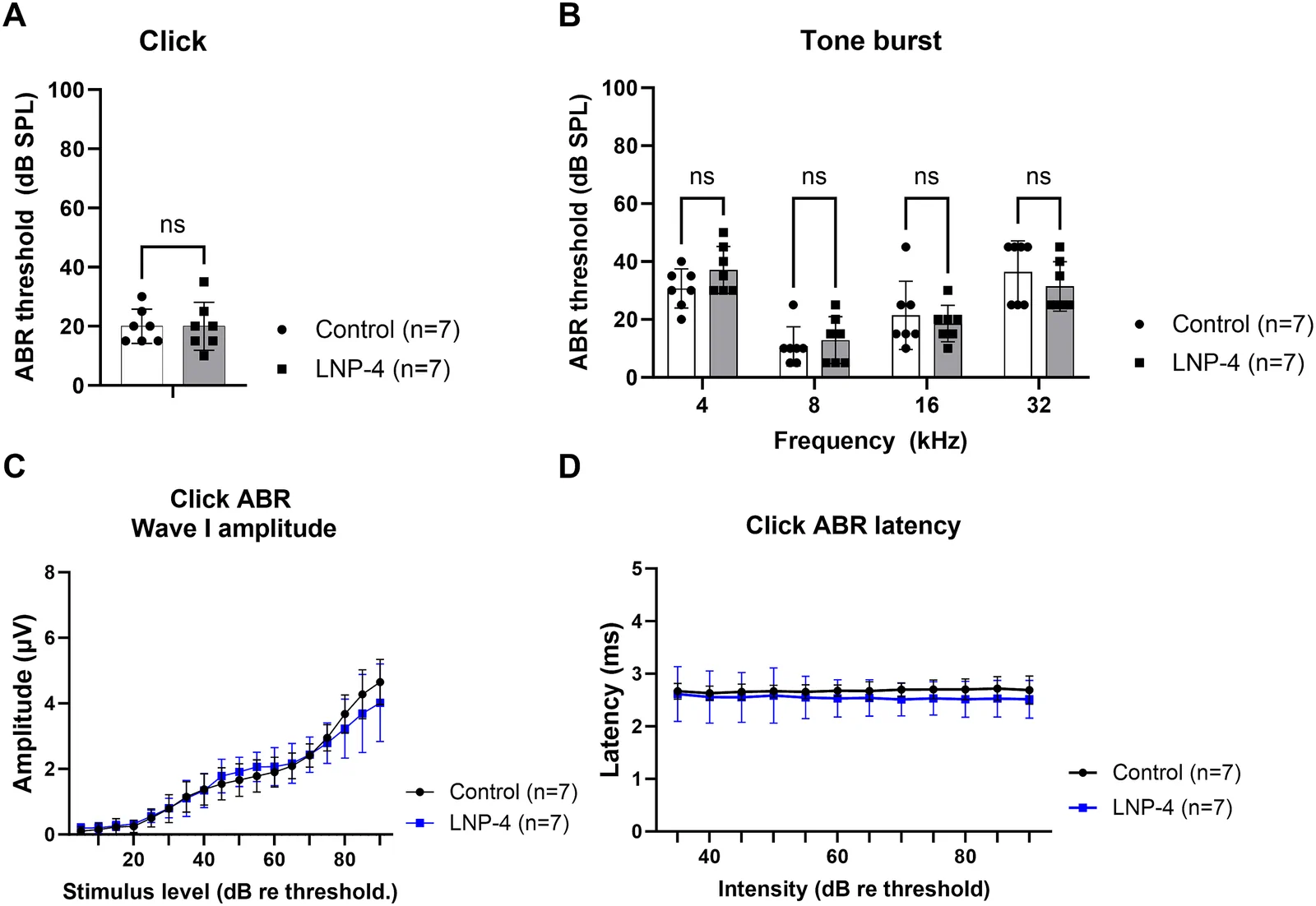
Auditory function and auditory neural responses following LNP administration. (A) Click-evoked ABR thresholds in control and LNP-treated mice (*n* = 7 per group). Thresholds were identical between groups, with no significant difference detected. (B) Tone-burst ABR thresholds measured at 4, 8, 16 and 32 kHz. No significant differences were observed between control and LNP-treated mice at any frequency. (C) Click-evoked ABR wave I amplitude as a function of stimulus level. Wave I amplitude increased with increasing stimulus level in both groups, with no significant differences between groups at any tested level. (D) Click-evoked ABR wave I latency across stimulus intensities. Wave I latency remained comparable between control and LNP-treated mice throughout the analyzed range. Data are presented as mean ± SEM. Click-evoked ABR thresholds were analyzed using an unpaired two-tailed Student's *t*-test. Tone-burst ABR thresholds, wave I amplitudes and wave I latencies were analyzed using two-way repeated-measures ANOVA with Geisser–Greenhouse correction, followed by Bonferroni's multiple-comparisons test. ns, not significant; *P* > 0.05.

Having established the safety profile of RWM delivery route, we next evaluated its transduction efficiency. Following RWM injection of mCherry–mRNA-LNPs, fluorescence imaging revealed robust mCherry expression in multiple cochlear structures, with particularly strong signals detected in the spiral ligament, scala vestibuli, scala tympani region, and spiral ganglia region. However, despite widespread transduction across perilymph-exposed regions, no detectable mCherry expression was observed in the hair cells in the organ of Corti (Fig. 5A–D").

**Fig. 5:**
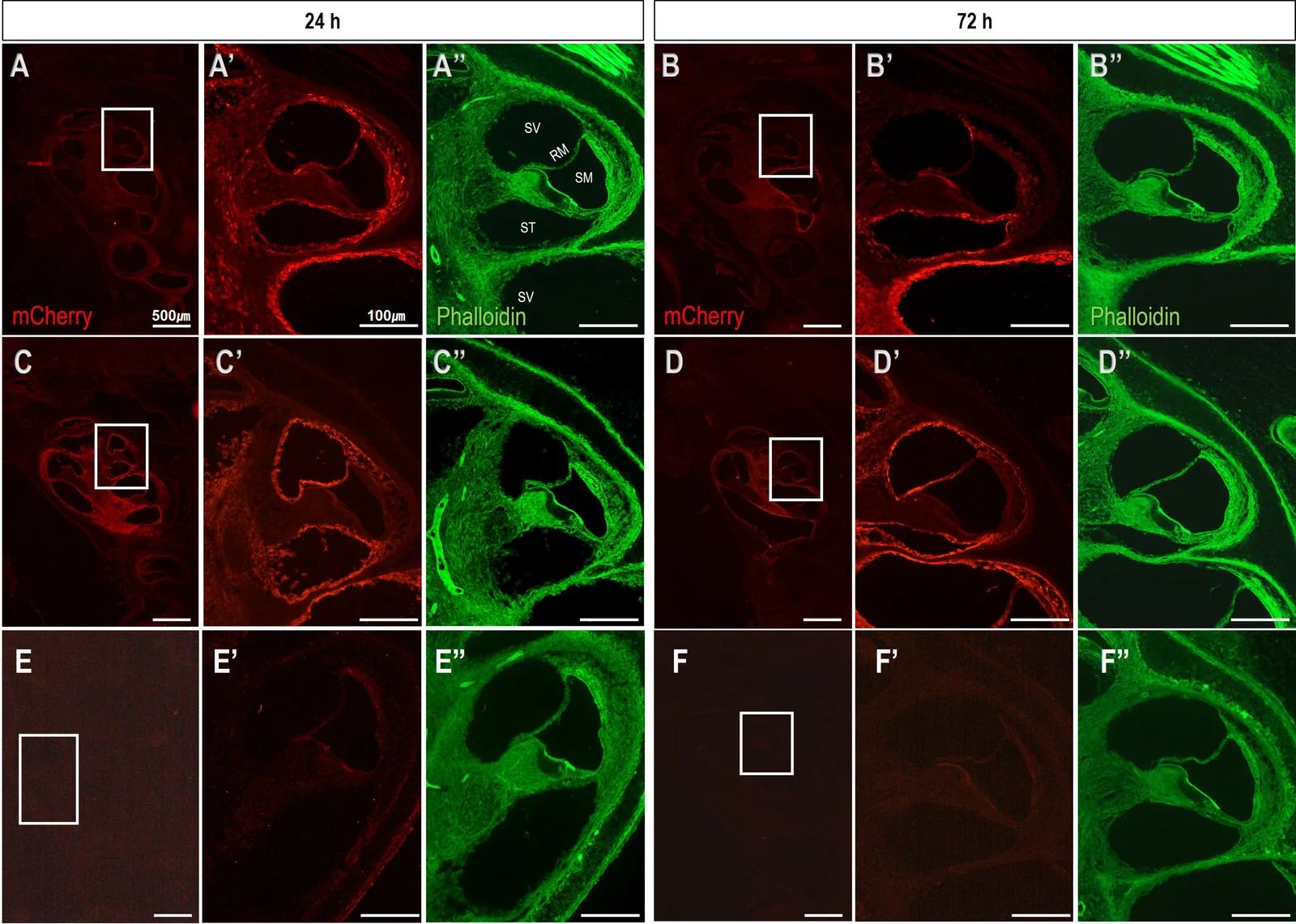
Time-dependent mCherry–mRNA LNP transduction in the mouse inner ear. (A, B) LNP was injected at P0, and mCherry expression was analyzed 24- and 72 h post-injection. (C, D) LNP formulated with 5% sucrose exhibited mCherry expression in perilymphatic regions, including the scala vestibuli and scala tympani. The white square marks the mid-apical region of the cochlear duct. Red fluorescence indicates mCherry expression, while green fluorescence represents phalloidin staining. (E–E') Negative control at 24 h, and (F–F′) 72 h. SV, scala vestibuli; RM, Reissner's membrane; SM, scala media; ST, scala tympani. Scale bars: 500 μm (A, B, C, D, E, F); 100 μm (A'–A", B′–B″, C′–C″, D′–D″). (For interpretation of the references to color in this figure legend, the reader is referred to the web version of this article.)

In this study, reporter-positive populations were characterized primarily by their anatomical location and distinctive cellular morphology in phalloidin-counterstained tissue sections, providing a confident regional map of LNP-mediated transduction across the cochlea. High-magnification orthogonal projections (Fig. 5A′–F′′) further support these regional assignments. The present experiments did not include cell type–specific markers, however, and position and morphology alone do not permit definitive identification of supporting cell subtypes, fibrocyte classes, macrophages, or spiral ganglion neurons; co-immunolabeling with established markers (*e.g.*, SOX2, PROX1, S100, CD68/IBA1, KCNJ10, and CTBP2) is required to establish cell type identity, and this is identified below as a limitation.

The identified transduction pattern is consistent with previous reports suggesting that the tight junctions of the reticular lamina may act as a physical barrier limiting nanoparticle diffusion into the endolymphatic space where hair cells reside, and that the comparatively high endocytic activity of fibrocytes in the spiral ligament may contribute to the preferential nanoparticle uptake in these cells. These mechanisms provide plausible explanations for the observed route-dependent transduction pattern; however, they were not directly examined in the present study, and therefore should be regarded as candidate mechanisms rather than experimentally established findings. Further studies incorporating approaches such as barrier-permeability manipulation and cell-specific assessment of nanoparticle uptake will be needed to determine their respective contributions.

Two further limits of the present dataset should also be noted. First, extracochlear tissues, including the brain and spinal cord, were not examined in this study. Because the cochlear aqueduct provides recognized communication between the perilymphatic space and the cerebrospinal fluid and is patent in the neonatal rodent, the absence of extracochlear tissue data precludes any conclusion that delivery was confined to the cochlea. Second, reporter expression was assessed only within the time intervals examined in this study; therefore, the duration of expression beyond intervals cannot be determined from the present data. A dedicated *in vivo* time-course study will be required to characterize the duration and persistence of reporter expression.

The primary objective of the present study was to characterize the regional distribution and transduction patterns of LNPs following different routes of inner-ear administration, rather than to establish the underlying mechanisms or the long-term duration of reporter expression. Within this scope, the consistency of the observed regional expression pattern was further examined in an *ex vivo* cochlear preparation. Following treatment of organ of Corti explants with mCherry-LNP (LNP-4) or mCherry-LNP containing 5% sucrose (LNP-4 with sucrose), mCherry expression was not detected in the hair cell layer but was clearly observed in the inner sulcus, outer sulcus, and supporting-cell-rich non-sensory regions (Fig. 6a–d and Fig. 6g–j). Additionally, localized mCherry expression was occasionally detected in regions corresponding to spiral ganglion cells (Fig. 6e and f). This expression pattern was stably maintained at both 72 h and 5 days after LNP treatment, and no substantial differences in expression intensity or distribution was observed between sucrose-treated and non-treated conditions (Fig. 6). These findings are consistent with the expression pattern observed *in vivo* (Fig. 5).

**Fig. 6:**
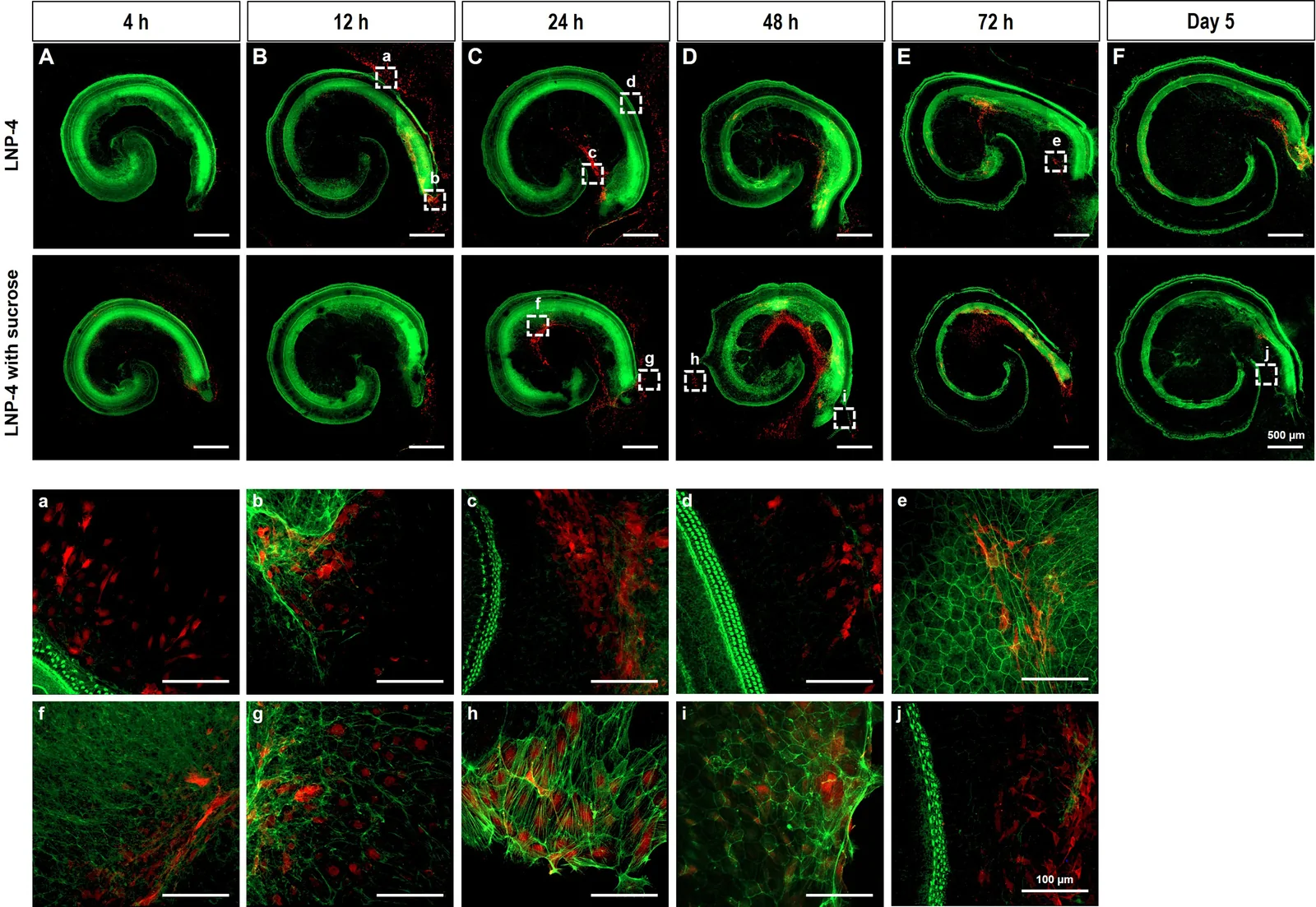
LNP-mediated delivery of mCherry mRNA to non-sensory regions of the mouse organ of Corti. Two groups of organotypic explants were treated with either non-sucrose LNP-mCherry (LNP-4) or 5% sucrose LNP-mCherry (LNP-4 with sucrose) to assess their ability to transfect cochlear cells at various time points. Confocal microscopy images of the explants were obtained using anti-mCherry and phalloidin immunostaining. The time points shown are (A) 4 h, (B) 12 h, (C) 24 h, (D) 48 h, (E) 72 h, and (F) day 5 in both LNP-4 and LNP-4 with sucrose-treated groups. Images are representative of at least three independent experiments (scale bar = 500 μm). Regions highlighted by white dashed boxes in panels A–F were magnified at (630×) and are shown in panels (a–j), with the scale bar for these high-magnification images at 100 μm.

To investigate whether direct administration into the endolymphatic compartment could improve hair cell transduction, we subsequently delivered mCherry–mRNA LNPs *via* scala media injection. This method resulted in a more confined expression pattern than the extensive expression observed using RWM administration, with mCherry fluorescence primarily localized to the cuticular plate in inner and outer hair cells and a subset of the outer sulcus region (Fig. 7). Notably, no mCherry expression was detected in the spiral ganglia or auditory nerve under the conditions examined. These findings indicate that scala media administration produced a more restricted transduction pattern, with limited detectable expression in regions beyond the endolymphatic compartment.

**Fig. 7:**
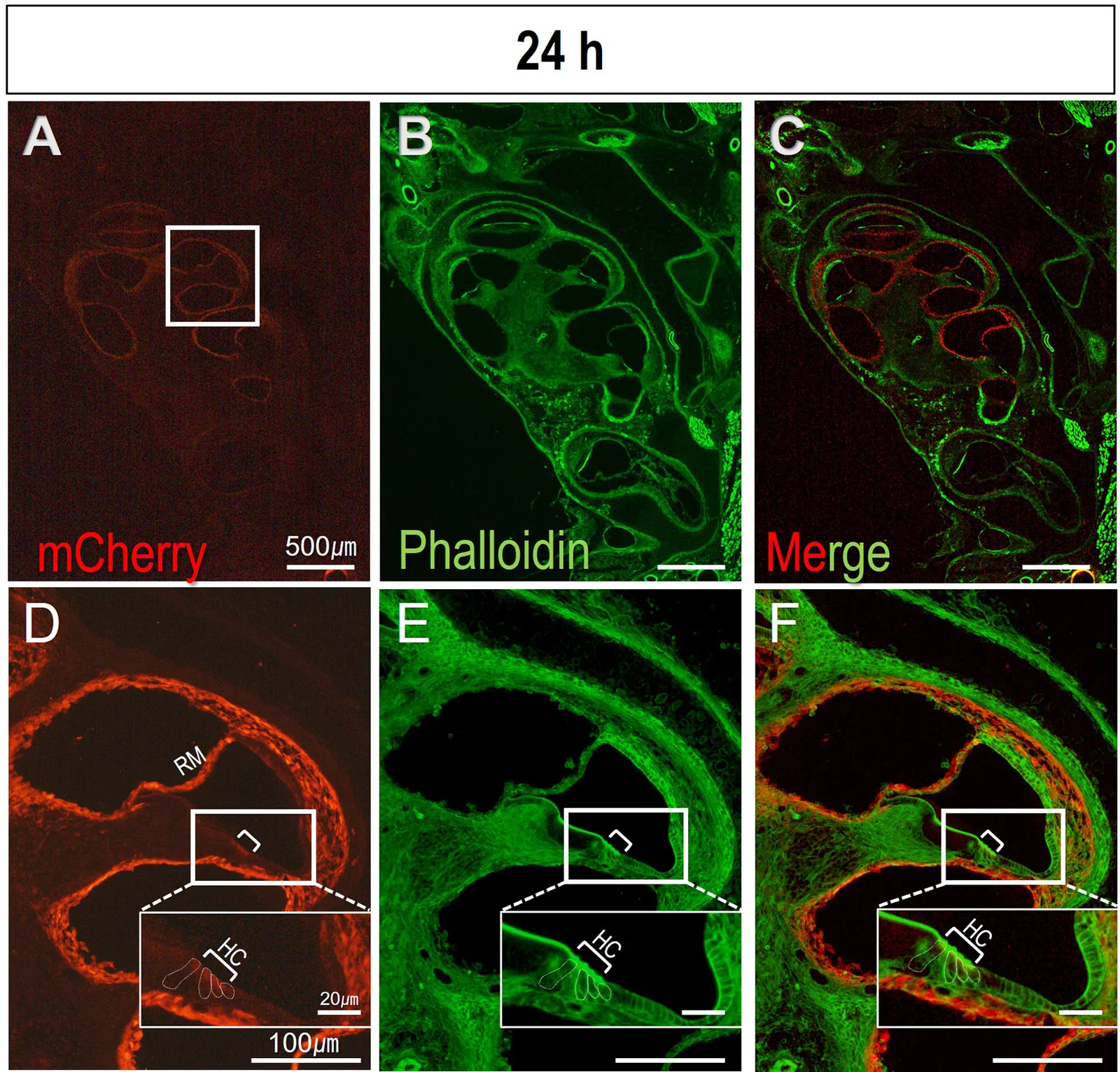
Limited transduction in the organ of Corti following scala media injection of mCherry–mRNA LNP. mCherry–mRNA LNP transduction following scala media administration at P0. (A–C) mCherry fluorescence indicates mCherry–mRNA LNP transduction, while phalloidin staining delineates the structure of the cochlear duct. (D–F) The magnified view depicts white brackets that clearly outline the boundaries of the hair cell region to provide a visual reference for its spatial organization. White dots mark the precise positions of inner and outer hair cells to facilitate their identification within the cochlear structure. RM, Reissner's membrane; HC, hair cell. Scale bars: 500 μm in A, B, C; 100 μm in D, E, F.

Collectively, these findings illustrate the distinct transduction profiles achieved through RWM and scala media administration. RWM delivery facilitates widespread expression throughout perilymph-exposed structures but largely excludes the organ of Corti. In contrast, scala media injection allows targeted delivery to the sensory epithelium but fails to achieve substantial gene transfer into hair cells beyond the cuticular plate. Because the two routes produce essentially non-overlapping distributions, the choice of route determines which cochlear compartments—and therefore which indications—are addressable, and this is the principal finding of the study.

Despite the direct exposure of hair cells to injected nanoparticles, robust intracellular mCherry expression was not observed, suggesting the presence of additional barriers to effective transduction. While the precise molecular mechanisms remain to be fully elucidated, several postulated mechanisms based on existing literature may account for this limited uptake: One potential factor is the relatively low endocytic activity of the apical surfaces of hair cells under physiological conditions, which may restrict the internalization of LNPs (Blanco et al., 2015). The extracellular matrix (ECM) can also be a physical barrier, impeding nanoparticle diffusion into the tissue and cellular uptake (Cahn et al., 2024), and structures such as the tectorial membrane partially shield outer hair cells; and macrophages, which are widely distributed in the lateral wall, basilar membrane, spiral ligament and stria vascularis, internalize nanoparticles through phagocytosis, often leading to accumulation in macrophage-rich regions (Hassett et al., 2024; Hayashi et al., 2020; Hirose et al., 2017; Hu et al., 2018; Liu and Xu, 2023). Although specific cell-type immunolabeling was not conducted to define exact cellular identities in this study, the anatomical distribution of mCherry signal aligns with these previously reported macrophage-rich regions. The literature-based insights offer plausible explanations for the observed LNP biodistribution within the inner ear.

### 3.5 Matching transient expression to developmentally restricted deafness genes

The transience of mRNA-mediated expression is often treated as a limitation of the modality. However, for a specific and clinically substantial class of deafness genes, it represents the appropriate pharmacological profile. These relevant genes share three characteristics: the functional protein is required only within a defined developmental window rather than continuously, it acts within non-sensory cells, and its action is non-cell-autonomous or structural, so that a transient pulse of protein produces a durable outcome. In this context, the spatial biodistribution mapped in the present study establishes a reference framework for identifying candidate genetic targets compatible with inner-ear LNP therapeutics.

*SLC26A4* serves as the anchor case regarding this paradigm. Using a doxycycline-inducible transgene on the *Slc26a4*-null background, the interval from E16.5 to P2 was defined as the period in which pendrin is required for the acquisition of normal hearing (the P0–P2 injection point used here lies within this interval); absence during this interval caused endolymphatic acidification, loss of the endocochlear potential, and failure to acquire hearing, whereas expression outside this period did not rescue hearing development (Choi et al., 2011). Moreover, pendrin is required to establish hearing but does not maintain it; meaning lifelong expression confers no advantage over one confined to the specified window. Notably, the challenge in pendrin gene therapy lies in spatial distribution rather than expression duration. In our earlier work (Kim et al., 2019), local *Slc26a4* delivery at P0–P1 rescued hearing at 4 weeks but failed to sustain it—a limitation attributed to insufficient transduction in the epithelial cells of the spiral prominence, root cells, and spindle cells of the stria vascularis. These critical niches correspond precisely to the compartments effectively targeted by the mRNA-LNPs evaluated in this study. While the exact transfection efficiency required for functional rescue remains an open question, the alignment between LNP biodistribution and pendrin-expressing niches highlights *SLC26A4* as a candidate worth exploring for transient mRNA therapeutics.

A second class comprises structural and secreted proteins that are made once and then persist. *STRC* (DFNB16, the second most frequent cause of autosomal recessive non-syndromic hearing loss after *GJB2*), *OTOA* (DFNB22), *OTOG*, *OTOGL*, *TECTA*, and *CEACAM16* are secreted by interdental cells, supporting cells, and the greater epithelial ridge during assembly of the tectorial membrane and stereociliary attachment structures and persist thereafter without ongoing synthesis in the same cells. A transient pulse of mRNA in the correct cells at the correct time could therefore yield a durable structural result. A third class concerns lateral wall and endocochlear potential development, in which *POU3F4* (DFNX2), *KCNQ1/KCNE1*, and the connexin network of the lateral wall act in mesenchyme-derived and marginal cell populations, and in which *GAS2* is required for the cytoskeletal architecture of pillar and Deiters' cells established during development (Casimiro et al., 2001; Kidokoro et al., 2014; Minowa et al., 1999; Song et al., 2011). These are the compartments reached after RWM administration.

However, certain prevalent targets such as *GJB2* (connexin 26) highlight the need for cell-type-specific refinement. Although supporting cells are within reach of this LNP formulation, connexin 26 functions within a spatially organized gap junction syncytium, and ectopic expression outside that network could perturb ionic coupling rather than restore it. Therefore, applying mRNA-LNPs to *GJB2* will likely require additional targeting strategies, such as cell-selective ligands or transcriptional control, rather than relying solely on untargeted delivery. However, incorporating actively targeted moieties onto nanoparticle platforms introduces significant regulatory and translational hurdles, including increased manufacturing complexity, batch-to-batch heterogeneity, and heightened chemistry, manufacturing, and controls (CMC) requirements for clinical approval.

Beyond spatial and cellular targeting, developmental timing represents another critical determinant for clinical translation. Unlike humans, mice are born with an immature auditory system and develop hearing postnatally, with auditory responses emerging around P12 (Wang et al., 2018). Therefore, the neonatal mouse should not be considered a direct developmental equivalent of the human newborn. Rather, the early postnatal mouse inner ear represents an immature developmental stage that is often considered developmentally analogous to the fetal human inner ear (Hastings and Brigande, 2020). This developmental window is characterized by active cochlear maturation and can provide a suitable model for evaluating inner-ear delivery and transduction during early auditory development. Nevertheless, differences in the timing of auditory maturation between mice and humans should be considered when extrapolating these findings to clinical applications.

## 4 Conclusion

In this study, we prepared a suitable mRNA-encapsulated LNP formulation and used it to characterize LNP-mediated mRNA delivery in the inner ear. LNPs were prepared using a systematic DoE approach leveraging CQAs and CPPs, and the resulting formulation was transferred to continuous-flow microfluidic manufacture without re-optimization. In HEI-OC1 cells, LNP-mediated delivery produced reporter expression in a larger and more uniformly distributed fraction of the population than Lipofectamine at a matched mRNA dose, with comparable total reporter protein and no detectable reduction in metabolic viability. *In vivo*, RWM injection produced widespread reporter expression in perilymph-exposed compartments, whereas scala media injection produced localized expression confined to the sensory epithelium and did not extend to the spiral ganglion region or auditory nerve; intracellular uptake by hair cells remained limited by either route. The two routes therefore give access to essentially non-overlapping sets of cochlear compartments, and this route dependence is the central finding of the study. These data provide the spatial map of mRNA-LNP biodistribution in the cochlea and the information required to design payload-specific studies: namely which compartments a given route can reach and which it cannot. These findings establish a spatial delivery framework that may facilitate the future investigation of therapeutic mRNA payloads in disease-relevant models. The observed distribution to the non-sensory epithelium, spiral ligament, and stria vascularis is of particular interest for deafness genes whose products are required transiently, in non-sensory cells, during a developmental window, and a functional study of LNP-delivered *Slc26a4* mRNA in the *Slc26a4*-null model is the planned next step. Given the established capacity of LNPs to encapsulate a wide range of RNA payload sizes—from relatively small mRNAs (∼1000 nt) to large mRNAs exceeding 10,000 nt—the formulation optimization and route-dependent spatial biodistribution framework established here provides a foundational benchmark for tailoring mRNA-LNP delivery to diverse therapeutic payloads and developing future mRNA-based inner ear therapeutics.

## CRediT authorship contribution statement

**Dongsik Park:** Writing – original draft, Visualization, Investigation. **Byeonghyeon Lee:** Writing – original draft, Visualization, Investigation. **Ji-Hyun Ma:** Visualization, Investigation. **Yeeun Kim:** Visualization, Investigation. **Jong Su Hwang:** Investigation, Data curation. **Hae Woo Lee:** Validation. **Myung Hoon Yoo:** Methodology, Data curation. **Un-Kyung Kim:** Writing – review & editing, Writing – original draft, Conceptualization. **Tae-Jun Kwon:** Writing – review & editing, Writing – original draft, Conceptualization.

## Ethics approval statement

All animal experiments were reviewed and approved by the IACUC of Kyungpook National University (Approval No. 2022–0147). No separate animal license number is issued by the institution.

All procedures were performed in accordance with the National Institutes of Health (NIH) Guide for the Care and Use of Laboratory Animals and complied with the ARRIVE 2.0 guidelines. Animals were housed under standardized environmental conditions and monitored throughout the study to ensure their health and welfare.

Neonatal mice undergoing RWM or scala media injection were anesthetized by hypothermia (ice anesthesia) before surgery. Mice used for ABR recordings were anesthetized by intraperitoneal administration of Alfaxalone and xylazine hydrochloride, as described in the Materials and Methods. At the experimental endpoint, all animals were euthanized by CO₂ inhalation in accordance with institutional animal welfare guidelines. P3 mice used for organotypic cochlear explant cultures were also euthanized by CO₂ inhalation before tissue collection.

Every effort was made to minimize animal suffering and distress, to reduce the number of animals used, and to apply the principles of 3Rs throughout the study.

## Ethics declaration

This study was conducted in accordance with the ARRIVE (Animal Research: Reporting of *In Vivo* Experiments) guidelines. This study was approved by the Kyungpook National University IACUC (Approval No. 2022–0147).

## Funding

This work was supported by the 10.13039/501100003725National Research Foundation of Korea (NRF) grant funded by the Korean government (MSIT) (grant number: No. 2022R1C1C1010262 to M.H.Y.; RS-2024-00406555 to U.K.K.; 2022R1A2C3003700 to U.K.K.) and a grant of the Korea Health Technology R&D Project through the 10.13039/501100003710Korea Health Industry Development Institute (KHIDI), funded by Ministry of Health & Welfare, Republic of Korea (grant number: RS-2022-KH130590 to U.K.K.)

## Declaration of competing interest

The authors declare that they have no known competing financial interests or personal relationships that could have appeared to influence the work reported in this paper.

## Data Availability

The data that support the findings of this study are available from the corresponding author upon reasonable request.
