# Supplementary material for: Exploration of biodistribution of mRNA-delivering lipid nanoparticles in the neonatal mouse inner ear
Source: Int J Pharm X. 2026 Sep 25;12:100675. doi: 10.1016/j.ijpx.2026.100675 (PMC13654544; doi:10.1016/j.ijpx.2026.100675)
Supplement: Supplementary file 1 [file mmc1.docx]

Supplementary material

**Exploration of Biodistribution of mRNA-Delivering Lipid Nanoparticles in the Neonatal Mouse Inner Ear**

*Dongsik Park, Byeonghyeon Lee, Ji-Hyun Ma, Yeeun Kim, Hae Woo Lee, Myung Hoon Yoo, Un-Kyung Kim and Tae-Jun Kwon*

**Figure S1.**

**Figure S1**. Diagnostic plots for the response surface models of LNP Critical Quality Attributes (CQAs). Normal probability plots of externally studentized residuals for (A) Particle Size, (C) Polydispersity Index (PDI), and (E) Encapsulation Efficiency (EE), demonstrating normal distribution of residuals along the reference line. Plot of predicted versus actual values for (B) Particle Size, (D) PDI, and (F) EE, demonstrating high predictive capability of the established models.

**Figure S2.**

**Figure S2**. Model and diagnostic plots for the response surface model of ln-transformed EGFP expression. (A) Three-dimensional (3D) response surface plots. (B) Contour plot. In the 3D response surface and contour plots, the color gradient from blue to green, yellow, and red represents increasing levels of ln-transformed EGFP expression. (C) Normal probability plot of externally studentized residuals for ln-transformed EGFP expression. (D) Plot of predicted versus actual values for ln-transformed EGFP expression. The diagnostic plots indicate that residuals were approximately normally distributed and that the predicted values were in good agreement with the experimentally observed values, supporting the adequacy of the fitted model.

**Figure. S3**

**Figure S3**. Comparison study of the size distribution of LNPs

**Figure. S4**

**Figure S4**. Size distribution of the mCherry-mRNA encapsulated LNPs (LNP-4) in PBS buffer and PBS buffer with 5 % sucrose.

**Table S1**. ANOVA results for reduced quadratic model evaluating the effects of parameters on the particle size. Model reduction was performed by eliminating statistically non-significant interaction and higher-order terms (AB and B^2^, p > 0.10) to improve model adequacy, and overall significance (p-value) while preserving model hierarchy. Statistical significance is indicated as follows: **p < 0.01, *p < 0.05, 0.05 ≤ p < 0.1 (marginal significance) and p ≥ 0.01 (not significant).

| Source | Sum of Squares | df | Mean Square | F-value | p-value |
| --- | --- | --- | --- | --- | --- |
| Model | 1734.42 | 3 | 578.14 | 8.91 | 0.0189* |
| A-Water/EtOH ratio | 483.30 | 1 | 483.30 | 7.45 | 0.0413* |
| B-N/P ratio | 503.43 | 1 | 503.43 | 7.76 | 0.0386* |
| A² | 747.68 | 1 | 747.68 | 11.53 | 0.0194* |
| Residual | 324.36 | 5 | 64.87 |  |  |
| Cor Total | 2058.78 | 8 |  |  |  |

Abbreviations: df, degree of freedom; F-value, Fisher's test statistic; p-value, probability value.

**Table S2**. Fit statistics for the particle size. Adequate precision values greater than 4 indicate an adequate signal-to-noise ratio, confirming that the developed regression model can be navigated across the design space.

| Fit statics | p-value |
| --- | --- |
| R² | 0.8424 |
| Adjusted R² | 0.7479 |
| Predicted R² | 0.5056 |
| Adequate Precision | 8.6841 |
| Std. Dev. | 8.05 |
| CV (%) | 7.53 |

Abbreviations: Std. Dev., Standard Deviation; CV, Coefficient of Variation

**Table S3**. ANOVA results for reduced quadratic model evaluating the effects of parameters on the PDI. Model reduction was performed by eliminating statistically non-significant interaction and higher-order terms (AB and B^2^, p > 0.10) to improve model adequacy, and overall significance (p-value) while preserving model hierarchy. Statistical significance is indicated as follows: **p < 0.01, *p < 0.05, 0.05 ≤ p < 0.1 (marginal significance) and p ≥ 0.01 (not significant).

| Source | Sum of Squares | df | Mean Square | F-value | p-value |
| --- | --- | --- | --- | --- | --- |
| Model | 0.1188 | 3 | 0.0396 | 198.65 | < 0.0001** |
| A-Water/EtOH ratio | 0.0845 | 1 | 0.0845 | 423.87 | < 0.0001** |
| B-N/P ratio | 0.0036 | 1 | 0.0036 | 17.82 | 0.0083** |
| A² | 0.0308 | 1 | 0.0308 | 154.27 | < 0.0001** |
| Residual | 0.0010 | 5 | 0.0002 |  |  |
| Cor Total | 0.1198 | 8 |  |  |  |

**Table S4**. Fit statistics for the PDI. Adequate precision values greater than 4 indicate an adequate signal-to-noise ratio, confirming that the developed regression model can be navigated across the design space.

| Fit statics | p-value |  |
| --- | --- | --- |
| R² | 0.9917 |  |
| Adjusted R² | 0.9867 |  |
| Predicted R² | 0.9699 |  |
| Adequate Precision | 30.9522 |  |
| Std. Dev. | 0.0141 |  |
| CV (%) | 7.17 |  |

Abbreviations: Std. Dev., Standard Deviation; CV, Coefficient of Variation

**Table S5**. ANOVA results for reduced quadratic model evaluating the effects of parameters on the EE. Model reduction was performed by eliminating statistically non-significant interaction and higher-order terms (AB and B^2^, p > 0.10) to improve model adequacy, and overall significance (p-value) while preserving model hierarchy. Statistical significance is indicated as follows: **p < 0.01, *p < 0.05, 0.05 ≤ p < 0.1 (marginal significance) and p ≥ 0.01 (not significant).

| Source | Sum of Squares | df | Mean Square | F-value | p-value |
| --- | --- | --- | --- | --- | --- |
| Model | 12773.73 | 3 | 4257.91 | 74.70 | 0.0001** |
| A-Water/EtOH ratio | 8551.13 | 1 | 8551.13 | 150.01 | < 0.0001** |
| B-N/P ratio | 46.54 | 1 | 46.54 | 0.8164 | 0.4077 |
| A² | 4176.07 | 1 | 4176.07 | 73.26 | 0.0004** |
| Residual | 285.02 | 5 | 57.00 |  |  |
| Cor Total | 13058.75 | 8 |  |  |  |

**Table S6**. Fit statistics for the EE. Adequate precision values greater than 4 indicate an adequate signal-to-noise ratio, confirming that the developed regression model can be navigated across the design space.

| Fit statics | p-value |  |
| --- | --- | --- |
| R² | 0.9782 |  |
| Adjusted R² | 0.9651 |  |
| Predicted R² | 0.9242 |  |
| Adequate Precision | 17.6853 |  |
| Std. Dev. | 7.55 |  |
| CV (%) | 12.37 |  |

Abbreviations: Std. Dev., Standard Deviation; CV, Coefficient of Variation

**Table S7**. GFP fluorescent intensity following transfection with EGFP-encoded mRNA delivered by LNPs from experimental runs

| Run | Independent factors (X) | | GFP Expression  (Green Fluorescent Intensity) |
| --- | --- | --- | --- |
|  | X_1_ | X_2_ |  |
| LN1 | 5 | 2 | 19606 |
| LN2 | 5 | 5 | 22584 |
| LN3 | 5 | 8 | 21534 |
| LN4 | 1 | 5 | 1975 |
| LN5 | 1 | 2 | 589 |
| LN6 | 3 | 5 | 31455 |
| LN7 | 3 | 8 | 46500 |
| LN8 | 1 | 8 | 3786 |
| LN9 | 3 | 2 | 8096 |

X_1_: aqueous to ethanol ratio, X_2_: N/P ratio

**Table S8**. ANOVA results for reduced quadratic model evaluating the effects of parameters on the natural log(ln)-transformed GFP expression. Model reduction was performed by eliminating statistically non-significant interaction and higher-order terms (B^2^, p > 0.10) to improve model adequacy, and overall significance (p-value) while preserving model hierarchy. Statistical significance is indicated as follows: **p < 0.01, *p < 0.05, 0.05 ≤ p < 0.1 (marginal significance) and p ≥ 0.01 (not significant).

| Source | Sum of Squares | df | Mean Square | F-value | p-value |
| --- | --- | --- | --- | --- | --- |
| Model | 16.55 | 4 | 4.14 | 40.20 | 0.0017** |
| A-Water/EtOH ratio | 9.83 | 1 | 9.83 | 95.50 | 0.0006** |
| B-N/P ratio | 2.28 | 1 | 2.28 | 22.20 | 0.0092** |
| AB | 0.7804 | 1 | 0.7804 | 7.58 | 0.0512 |
| A² | 3.66 | 1 | 3.66 | 35.53 | 0.0040** |
| Residual | 0.4117 | 4 | 0.1029 |  |  |
| Cor Total | 16.97 | 8 |  |  |  |

**Table S9**. Fit statistics for the ln-transformed GFP expression. Adequate precision values greater than 4 indicate an adequate signal-to-noise ratio, confirming that the developed regression model can be navigated across the design space.

| Fit statics | p-value |  |
| --- | --- | --- |
| R² | 0.9757 |  |
| Adjusted R² | 0.9515 |  |
| Predicted R² | 0.8572 |  |
| Adequate Precision | 18.0155 |  |
| Std. Dev. | 0.3208 |  |
| CV (%) | 3.51 |  |

Abbreviations: Std. Dev., Standard Deviation; CV, Coefficient of Variation

**Table S10**. Manufacturing process and condition for LNP-1through LNP-3

| Materials | Ionizable lipids in formulation | Manufacturing process |
| --- | --- | --- |
| LNP-1 | SM-102 | Mixing in small volume (200 µL) |
| LNP-2 | ALC-0315 | Mixing in small volume (200 µL) |
| LNP-3 | SM-102 | Microfluidic mixing (2 mL) |

**Table S11**. Physicochemical properties of LNP-3 and LNP-4 associated with CQA.

| Materials | Mean particle size (nm) | PDI | EE (%) |
| --- | --- | --- | --- |
| LNP-3 | 50.2 | 0.12 | 95.8 |
| LNP-4 | 57.4 | 0.10 | 97.8 |
| LNP-4 with sucrose | 67.7 | 0.09 | 97.8 |
